# Janus Ag/Fe-HfO_2_ nanoparticles for enhanced radio-photothermal tumor therapy via magnetic resonance imaging and multienzyme activity

**DOI:** 10.1016/j.mtbio.2025.102083

**Published:** 2025-07-11

**Authors:** Xinying Liu, Baohui Liu, Conglong Chen, Chaowei Hong, Jia Xu, Yixuan Ruan, Ling Huang, Shanni Hong, Wei Chen, Ye Kuang

**Affiliations:** a Fujian Key Laboratory of Drug Target Discovery and Structural and Functional Research, School of Pharmacy, Fujian Medical University, Fuzhou, 350122, China; b School of Basic Medical Sciences, Fujian Medical University, Fuzhou, 350122, China; c School of Medical Imaging, Fujian Medical University, Fuzhou, 350122, China

**Keywords:** Radiotherapy, Photothermal therapy, Janus nanoparticles, Nanozyme, Magnetic resonance imaging, Multifunctional nanoradiosensitizer

## Abstract

A significant challenge in modern radiotherapy (RT) is to minimize the detrimental effects on adjacent healthy tissues while concurrently increasing the susceptibility of tumor tissue to radiation. Investigating nanoradiosensitizers with multifunctional properties has a lot of potential as a highly effective strategy for augmenting the efficacy of RT. A novel Janus Ag/Fe-HfO_2_ nanoparticle was synthesized in a single-step process. This versatile nanoradiosensitizer combines photothermal capabilities, enzyme-like activities, and the capacity for magnetic resonance imaging (MRI). The surfaces have been meticulously engineered with PEG to boost biocompatibility and adorned with RGD-targeting peptides that selectively target tumor cells (Ag/Fe-HfO_2_-PEG-RGD). The characterization results have demonstrated the successful preparation of Janus Ag/Fe-HfO_2_ nanoparticles with a diameter of roughly 10 nm. Furthermore, the photothermal properties, MRI ability, and enzymatic catalytic activities (generate O_2_, produce hydroxyl radical, and consume GSH) of Ag/Fe-HfO_2_-PEG-RGD were verified *in vitro* experiments. *In vivo* experiments, by integrating *T*_*1*_-weighted MRI-guided RT with the enzymatic activities of these nanoparticles, along with the application of photothermal heating, we have achieved a synergistic enhancement in the therapeutic efficacy of RT. Moreover, it stimulates tumor-specific immune responses, leading to an increased presence of tumor-infiltrating CD8^+^ T cells. This integrated multimodal nanotherapeutic platform, which capitalizes on the potent enhancement of RT effects, offers a promising new strategy in the ongoing battle against cancer.

## Highlights

• Single-step synthesis of Janus Ag/Fe-HfO_2_ nanoparticles with multifunctional properties.
• Showed that Janus Ag/Fe-HfO_2_ have photothermal, MRI, and enzyme-like activities.
• Achieved synergistic enhancement of RT efficacy via MRI-guided RT and PTT *in vivo*.
• Triggered tumor-specific immune responses and boosted CD8^+^ T cells.

## Graphical abstract

**Figure:**
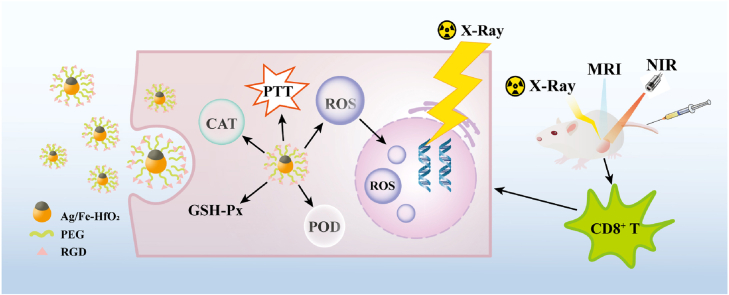

## 1 Introduction

Malignant neoplasms constitute a grave risk to human well-being, with roughly 70 % of individuals affected by cancer relying on radiotherapy (RT) as an integral component of their comprehensive treatment regimens in clinical settings [1,2]. RT is an essential component of the standard clinical treatment for cancer, often employed as a standalone treatment or in conjunction with other therapeutic modalities [3]. RT primarily relies on the application of high-dose ionizing radiation to either directly disrupt DNA or indirectly destroy cancer cells by generating cytotoxic free radicals. The extent of DNA damage caused by this irradiation is linked to the amount of energy deposited at the location of tumors. However, as tumors are soft tissues with a low radiation attenuation factor, a significant radiation dose is required to effectively target the tumor, which unfortunately can also result in collateral damage to the adjacent healthy tissues. Consequently, a major obstacle in contemporary RT is the need to mitigate the adverse effects on surrounding healthy tissues with the enhancement of tumor tissue sensitivity to radiation. Moreover, due to the presence of inherent or therapy-induced radio resistance, solitary RT sessions are often insufficient to achieve the desired level of tumor suppression. To address this fundamental issue, a variety of therapeutic approaches, including chemotherapy (CHT), chemodynamic therapy (CDT), photothermal therapy (PTT), photodynamic therapy (PDT), and immunotherapy (IT), have been investigated for their potential to enhance the effects of RT in both laboratory studies and clinical settings [[4], [5], [6], [7], [8]]. In addition, advancements in bioimaging are pivotal for acquiring critical biological insights, including the dimensions and shape of tumors, which are essential for refining targeted treatment approaches and reducing the damage to normal tissue [9].

Thanks to the swift advancements in material science and nanotechnology, a multitude of radiosensitizers have been applied in practice and have achieved excellent results in enhancing the effects of RT [[10], [11], [12]]. Among them, high-Z nano-systems, due to their larger X-ray attenuation coefficients, are extensively researched as the next generation of radio-sensitizing agents. These nano-systems capture X-rays, leading to the emission of photoelectrons, Compton electrons, or Auger electrons. Subsequently, these electrons engage with biomolecules and water molecules to produce cytotoxic free radicals, which in turn amplify the impact of RT. Among these, Hafnium (Hf), as a non-toxic chemically inert material, has made some progress in the fields of biosensing and X-ray contrast agents. As a high-Z element, Hf can emit secondary electrons and photoelectrons in response to X-rays, exhibiting a strong ionization effect and playing a role in enhancing RT [1,3]. Moreover, nano-RT sensitizers based on Hafnium oxide (HfO_2_) nanoparticles, such as NBTXR3, are currently undergoing clinical trials (clinical trial numbers NCT01946867 and NCT02901483), and the latest research has proven that this material has good biocompatibility [13,14]. Therefore, the construction of multifunctional nano-RT sensitizers based on HfO_2_ nanoparticles can effectively enhance the efficacy of RT and has good potential for clinical translation.

The tumor microenvironment (TME) exacerbates hypoxia in tumors, and increases acidity and glutathione (GSH) levels, thereby significantly diminishing the effectiveness of RT. Numerous metal nanoparticles possess multi-enzymatic capabilities that can modulate TME at the cellular level [15]. Nanozyme with catalase-like (CAT) activity can convert endogenous hydrogen peroxide (H_2_O_2_) into oxygen (O_2_), reducing hypoxia in the TME and enhancing the effectiveness of RT. Additionally, the activity of glutathione peroxidase (GSH-Px) converts GSH into its oxidized form (GSSH), lowering GSH levels within tumor cells and further disrupting the redox balance in the TME, which is advantageous for RT. Consequently, the enzymatic properties of metal nanomaterials can ameliorate tumor hypoxia and address the limited efficacy of RT, while also generating substantial reactive oxygen species (ROS) during RT, leading to cancer cell necrosis and apoptosis [16]. Studies indicate that ultra-small Fe_3_O_4_ nanoparticles possess *T*_*1*_-weighted MRI imaging capabilities, making them promising candidates for Fe doping in RT sensitizers, offering benefits such as multi-enzymatic activity, low toxicity, and MRI-guided treatments [7,17,18].

Silver nanoparticles (Ag NPs) have found widespread use in diverse biomedical fields, leveraging their inherent properties such as antiviral, antimicrobial, antifungal, antiangiogenic effects, anti-inflammatory, facilitation of tissue repair, and anticancer activity [[19], [20], [21]]. Recently, numerous research teams have showcased the therapeutic effectiveness of Ag NPs, capitalizing on their inherent properties to curb the growth and trigger the apoptosis of cancer cells [22,23]. Moreover, the distinctive surface plasmon resonance (SPR) characteristics of Ag NPs make them ideal candidates for PTT. This process involves the conversion of absorbed laser photons into thermal energy, leading to an increase in temperature. Silver and gold with plasmonic properties exhibit superior photostability and more robust light absorption capabilities due to their distinctive SPR, surpassing the performance of traditional photo absorbing dyes. Given the superior SPR efficiency of silver, Ag NPs emerge as a viable and potentially superior alternative [22,24,25].

**Scheme 1:**
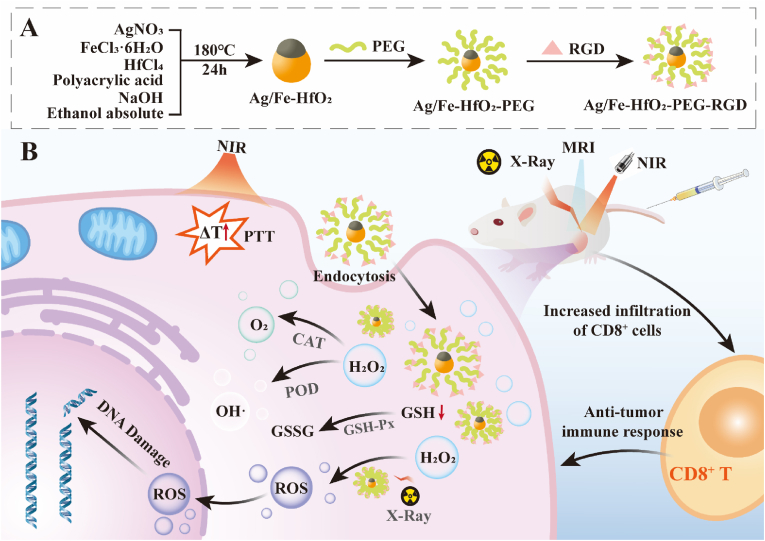
Overview of the fabrication and tumor-inhibiting action of Ag/Fe-HfO_2_-PEG-RGD. (A) Depiction of the fabrication process for Ag/Fe-HfO_2_-PEG-RGD. (B) A diagrammatic representation of Ag/Fe-HfO_2_-PEG-RGD integrated with MRI-guided RT/PTT/enzyme catalytic activity for a synergistic therapeutic approach.

Therefore, a novel Janus Ag/Fe-HfO_2_ NPs was produced by a one-step synthesis that acts as a multifunctional nano-RT sensitizer with PTT effect, enzymatic-like activity, and MRI. This breakthrough successfully resolves the key shortcomings of conventional RT by merging multiple functionalities into an integrated system. The surface of these nanoparticles is modified with PEG that enhances biocompatibility and RGD targeting peptides that aim at tumor cells (Ag/Fe-HfO_2_-PEG-RGD) (Scheme 1A). As shown in Scheme 1B, the construction of this nanosystem facilitates the precise delivery of nano-RT sensitizers in the body. *T*_*1*_-weighted MRI-guided RT, coupled with the enzymatic activity of nanoparticles to combat hypoxia, generate the hydroxyl radicals and diminish GSH levels, as well as photothermal heating, collectively operate synergistically to amplify the therapeutic impact of RT. This multifaceted approach leads to significant DNA damage, thereby enhancing the efficacy of cancer treatment. Simultaneously, the approach stimulates tumor-specific immune responses, resulting in an elevated presence of CD8^+^ T cells within the tumor tissue. This strategic intervention ultimately aims to effectively eradicate tumor cells and prevent their resurgence, thereby achieving a more durable therapeutic outcome.

## 2 Experimental section

### 2.1 Materials

Hafnium (IV) chloride (HfCl_4_) was purchased from Thermo Scientific. Iron chloride hexahydrate (FeCl_3_·6H_2_O), and silver nitrate (AgNO_3_) were purchased from Aladdin. Sodium hydroxide (NaOH), Ethanol absolute (≥99.7 %) were purchased from TCI Development Co. Ltd. Polyacrylic Acid (PAA), Amine-PEG-CH_2_COOH (MW 2000), 1-(3-Dimethylaminopropyl)-3-ethylcarbodiimide hydrochloride (EDC) and Tris (4,7-diphenyl-1,10-phenanthroline) ruthenium (II) dichloride (RDPP) were purchased from Bidepharm. cRGDfk(cyclo-(Arg-Gly-Asp-D-Phe-Lys)) was purchased from GL Biochem (Shanghai) Ltd. 2′,7′-dichlorodihydrofluorescein diacetate (DCFH-DA), AM-PI Dyeing kit, Hoechst and 4′,6-diamidino-2-phenylindole (DAPI), γ-H2AX DNA Damage kit, Crystal violet dye and paraformaldehyde were purchased from Beyotime Biotechnology (Shanghai) Co., Ltd. Phosphate buffered saline (PBS), and RPMI-1640 medium were purchased from Sangon Biotech (Shanghai) Co. Ltd. Fetal bovine serum (FBS) was purchased from ExCell. All chemicals and solvents were purchased from commercial sources and employed without additional purification.

### 2.2 Synthesis of Ag/Fe-HfO_2_ NPs

Initially, the Janus Ag/Fe-HfO_2_ NPs were synthesized via a one-pot process. HfCl_4_, AgNO_3_, FeCl_3_•6H_2_O, and PAA were dissolved in ethyl alcohol and mixed thoroughly at room temperature. Subsequently, 0.1 g of NaOH was dissolved in 10 mL of anhydrous ethanol and slowly added to a round-bottomed flask, with continuous stirring for an additional 30 min. After stirring, the solution was poured into a hydrothermal synthesis system and kept at a temperature of 180 °C for a period of 24 h. Once the reaction was complete, the product was centrifuged at 10,000 rpm for 10 min, and subsequently, the supernatant was carefully removed. The precipitate was then washed three times with anhydrous ethanol to remove any residual impurities. The purified precipitate was subsequently dissolved in water and underwent final centrifugation at 10,000 rpm to eliminate any large and medium-sized particles present in the solution. The purified solution was then freeze-dried in a vacuum freeze-dryer to yield the Janus Ag/Fe-HfO_2_ NPs sample powder. The powder was then resuspended in water to prepare a solution at a concentration of 10 mg/mL for subsequent use.

### 2.3 Synthesis of Ag/Fe-HfO_2_-PEG-RGD

Initially, 1 mL of Janus Ag/Fe-HfO_2_ NPs solution (10 mg/mL) was combined with 1 mL of deionized water in a small beaker. Then, EDC was introduced to the mixture and stirred for 30 min to activate the carboxyl groups present on the PAA. Subsequently, 5 mg of Amine-PEG-CH_2_COOH was dissolved in the solution, and magnetic stirring was continued for 8 h to facilitate the conjugation reaction. Following this, 200 μL of cRGDfk (at a concentration of 5 mg/L) was added to the solution, and stirring was maintained for an additional 8 h to ensure thorough binding. Finally, the conjugated product was purified by dialysis against deionized water to remove any unreacted impurities.

### 2.4 Characterization

The size and shape of the nanoparticles were examined through High-Resolution Transmission Electron Microscopy (HRTEM). UV–Vis–NIR spectroscopy, Fourier-transform infrared spectroscopy (FTIR), X-ray diffraction (XRD), and X-ray photoelectron spectroscopy (XPS) were utilized to determine the elemental composition and detailed structure of the nanoparticles. The hydrodynamic diameter and zeta potential were determined using a particle size and zeta potential analyzer. The concentrations of iron (Fe) and hafnium (Hf) within the nanoparticles were measured by Inductively Coupled Plasma Mass Spectroscopy (ICP-MS). The magnetic resonance relaxivity and *T*_*1*_-weighted imaging properties were evaluated with a GY-PNMR-10 pulsed nuclear magnetic resonance imaging system, supplied by Shanghai Huan Tong Science and Education Equipment Co. Ltd.

### 2.5 Oxygen dissolution

To assess the oxygen-generating capacity of the Janus Ag/Fe-HfO_2_ NPs, we evaluated the oxygen production in solutions of varying concentrations (0, 50, 100, and 200 μg/mL) employing a dissolved oxygen meter from Mettler Toledo. This measurement was crucial for understanding the potential to enhance oxygen levels in biological environments.

### 2.6 Photothermal performance test

Janus Ag/Fe-HfO_2_ NPs solutions with varying concentrations (0, 1.25, 2.5, 5, and 10 mg/mL) were aliquoted into a 96-well plate and exposed to an 808 nm near-infrared (NIR) laser at a power density of 1 W/cm^2^ for 5 min. Temperature elevation was monitored at 10-s intervals using a thermometer. Furthermore, the photothermal conversion stability of the Janus Ag/Fe-HfO_2_ NPs was examined under identical experimental conditions. A 5 mg/mL sample was irradiated for 5 min, after which the 808 nm laser was deactivated, allowing the sample to cool naturally to room temperature. Subsequently, four cycles of on/off irradiation were conducted, with temperature changes being recorded throughout. Under 808 nm laser irradiation, a 5 mg/mL Janus Ag/Fe-HfO_2_ NPs solution was subjected to different power densities, namely 0.5, 1, and 1.5 W/cm^2^. Infrared thermal imaging (using the TiS55+ from Fluke Company, USA) was employed to capture thermal images and document the temperature variations at different time points.

And the photothermal conversion efficiency (*η*) could be calculated below (1)–(3):

(1) $$\begin{array}{c} \eta =\frac{hS\left(T_{\max}‐T_{surr}\right)‐Q_{dis}}{I\left(1‐10^{{‐A}_{\lambda }}\right)} \end{array}$$

(2) $$\begin{array}{c} Q_{dis}=\frac{mC_{water}\left(T_{\max\left(water\right)}‐T_{surr}\right)}{\tau_{water}} \end{array}$$

(3) $$\begin{array}{c} hS=\frac{mC_{water}}{\tau } \end{array}$$

where *h* is the heat transfer coefficient, *S* is the surface area of the sample cell, *T*_max_ is the final temperature during the heating process, *T*_*surr*_ is the surrounding temperature, *I* means the power density of the irradiated laser, *A*_*λ*_ is the absorbance of Ag/Fe-HfO_2_ at 808 nm, and *m* is the quality of the solution, *C*_*water*_ represents the specific heat capacity of water. *θ* is calculated according to formula (4):

(4) $$\begin{array}{c} \theta =\frac{T-T_{surr}}{T_{\max}-T_{surr}} \end{array}$$

profile the variation of *t* with *τln(θ)* to obtain the slopes *τ*. In this way, the *η* of Ag/Fe-HfO_2_ NPs was calculated.

### 2.7 Glutathione peroxidase (GSH-Px)-like activity of Ag/Fe-HfO_2_

Janus Ag/Fe-HfO_2_ NPs at concentrations of 0, 0.5, 1.25, 2, and 5 mg/mL were suspended in 2 mL of phosphate-buffered saline (PBS, 0.1 M, pH 6.8) that had been enriched with glutathione (GSH, 0.15 mM). The mixture was permitted to react for a total of 4 h. Meanwhile, 5 mg/mL Ag/Fe-HfO_2_ NPs was allowed to react for 0, 0.5, 1, 2, 4, and 6 h under the aforementioned conditions. Subsequently, DTNB was introduced to achieve an ultimate working concentration of 300 μg/mL. The optical absorption intensity at 412 nm was measured at the culmination of the chromogenic reaction. This assay was designed to evaluate the reactivity of the nanoparticles with GSH under acidic conditions, providing insights into their potential redox activity.

### 2.8 ·OH generation

The absorption spectra in the ultraviolet–visible (UV–Vis) range were documented following the addition of 1 mM hydrogen peroxide (H_2_O_2_) to solutions containing varying concentrations of Janus Ag/Fe-HfO_2_ NPs (0, 0.5, 1.25, 2, and 2.5 mg/mL) in a 3,3′,5,5′-tetramethylbenzidine (TMB) substrate solution at a concentration of 0.5 mM. This procedure was employed to assess the catalytic activity of the nanoparticles in the presence of a peroxidase substrate, providing valuable information on their potential for catalytic applications. The ·OH-generating activity of Ag/Fe-HfO_2_ NPs was further verified by ESR spectroscopy using DMPO as a probe. In brief, 20 μL DMPO, 20 μL Ag/Fe-HfO_2_ NPs (100 μg/mL), and 20 μL H_2_O_2_ (5 mM) were added to 40 μL PBS at pH = 5.5, respectively. Then, the mixture was exposed to ESR to capture characteristic 1: 2: 2: 1 ·OH signal.

To determine the Michaelis–Menten kinetics of Ag/Fe-HfO_2_ NPs, Ag/Fe-HfO_2_ NPs (100 μg/mL), TMB (0.8 mM), and different concentrations of H_2_O_2_ (0.2, 0.5, 1, 1.5, and 2 mM) were added to PBS at pH = 5.5, respectively. The Michaelis–Menten kinetic curve was acquired by plotting the initial velocity against H_2_O_2_ concentration, and the V_max_ and K_m_ were calculated by Lineweaver–Burk plot.

### 2.9 ROS generation in solution

Samples of Janus Ag/Fe-HfO_2_ NPs with varying concentrations were prepared in an aqueous solution, ensuring a final concentration of 20 μM for the DCFH-DA probe and 100 μM NaOH. The fluorescence intensity, characterized by excitation at 450 nm and emission at 500 nm, was measured after exposing the solutions to X-ray doses of 0, 2, 4, 6, or 8 Gy, respectively. This experimental setup was designed to evaluate the ROS generation capacity of the Janus Ag/Fe-HfO_2_ NPs under ionizing radiation, which is crucial for understanding their potential applications in RT.

### 2.10 In vitro MRI performance

The MRI performance of Janus Ag/Fe-HfO_2_ NPs and Ag/Fe-HfO_2_-PEG-RGD was assessed using a scanning MRI system. Aliquots of 200 μL of the samples, prepared at various concentrations (0.25, 0.50, 1.00, 2.00, and 4.00 mM), were placed in a 96-well plate. The elemental concentration of iron was ascertained using Inductively Coupled Plasma Mass Spectrometry (ICP-MS). The relaxivity, r_1_, was calculated by fitting the curve of the inverse of the *T*_*1*_ relaxation time (1000/*T*_*1*_) against the iron ion concentration. The slope of the fitted line represented the r_1_ value. The parameters for *T*_*1*_-weighted MRI were configured with an echo time (TE) of 20 ms and a repetition time (TR) of 3000 ms. These parameters were essential in assessing the nanoparticles’ efficacy as *T*_*1*_-contrast agents for MRI.

### 2.11 Cell culture and cytotoxicity assay

Mouse breast carcinoma cells (4T1 cells) and mouse mammary epithelial cells (HC11 cells) were grown in RPMI 1640 medium enriched with 10 % fetal bovine serum (FBS), streptomycin at a concentration of 100 μg/mL, and penicillin at 100 units/mL, under conditions of 5 % CO_2_ at a temperature of 37 °C. The cytotoxic effects of Janus Ag/Fe-HfO_2_ NPs and Ag/Fe-HfO_2_-PEG-RGD were evaluated using the MTT assay. For this, cells (150 μL at a density of 3 × 10^4^ cells/mL) were seeded into 96-well plates and exposed to NPs at various concentrations (0, 6.25, 12.5, 25, 50, 100, or 150 μg/mL) for 24 h. Subsequently, 50 μL of MTT solution (5 mg/mL) was added, and the cells were incubated for an additional 4 h. Afterward, the cells were rinsed with PBS to remove the NPs, and 100 μL of DMSO was added to dissolve the formazan crystals that had precipitated in each well. The absorbance at 490 nm was measured using a microplate spectrophotometer (Epoch 2, Biotek, USA). This assay was conducted to assess the viability of the cells in response to the treatment with the nanoparticles. Furthermore, to investigate the induction of apoptosis by Janus Ag/Fe-HfO_2_ NPs and Ag/Fe-HfO_2_-PEG-RGD, additional experiments were conducted.

### 2.12 Intracellular uptake performance

The cellular uptake of Janus Ag/Fe-HfO_2_ NPs and Ag/Fe-HfO_2_-PEG-RGD was visualized using an inverted fluorescence microscope. The 4T1 cells were cultured in 6-well plates, and subsequently, 2 mL of FITC-labeled Janus Ag/Fe-HfO_2_ NPs and Ag/Fe-HfO_2_-PEG-RGD solutions (100 μg/mL of Hf) were introduced for incubation periods of 2, 4, 6, and 12 h. After incubation, the cells were stained with DAPI solution (3 μg/mL, with 1 mL per well) to counterstain the nuclei. This staining procedure facilitated the observation of both the cellular nuclei and the internalized nanoparticles, allowing for the assessment of the uptake efficiency and intracellular distribution over time.

### 2.13 Intracellular O_2_ generation and detection

The generation of intracellular oxygen was assessed using the trapping agent [Ru(dpp)_3_]Cl_2_. The 4T1 cells were grown in 6-well plates, and an anoxic model was induced by treatment with CoCl_2_. The cells were then sequentially incubated with a solution of Ru[(dpp)_3_]Cl_2_ (8 μg/mL) and varying concentrations of Ag/Fe-HfO_2_-PEG-RGD (0, 6.25, 12.5, 25, 50, and 100 μg/mL). Following incubation, the cellular sections were examined using an inverted fluorescence microscope to capture fluorescence images. This methodology enabled the visualization and quantification of oxygen production within the cells, which is vital for gauging the capacity to mitigate hypoxia and boost the potency of cancer treatments.

### 2.14 Calcein-AM and propidium iodine (PI) staining assay

4T1 cells were plated in 6-well plates and cultured with fresh medium, as well as Ag/Fe-HfO_2_-PEG-RGD (100 μg/mL of Hf) under various conditions: PBS, RT, Ag/Fe-HfO_2_-PEG-RGD alone, Ag/Fe-HfO_2_-PEG-RGD with RT, Ag/Fe-HfO_2_-PEG-RGD with PTT and Ag/Fe-HfO_2_-PEG-RGD with RT and PTT. Unless specified otherwise, the drug concentrations used in subsequent cell experiments are as described above. Following a 24-h incubation, the cells were rinsed, collected, and stained with calcein-AM/PI to evaluate cell viability and membrane integrity. The stained cells were then subjected to high-speed CLSM for imaging. This approach allowed for the visualization of cellular uptake and the effects of the nanoparticles on cell viability under different treatment conditions.

### 2.15 In vitro ROS generated

The generation of reactive ROS by the nanoparticles was investigated using DCFH-DA assays. 4T1 cells were plated onto confocal dishes and allowed to grow for 12 h before being exposed to various treatment conditions with cell medium. The cells were then gently washed twice with PBS and incubated with the DCFH-DA probe at 37 °C for 20 min to detect ROS. After three additional washes in PBS to remove the excess probe, the cells were analyzed using CLSM to visualize the fluorescence indicative of ROS production. Additionally, the measurement of ROS levels was conducted via flow cytometry, offering a more accurate and quantitative assessment of the oxidative stress caused by the NPs within the 4T1 cells.

### 2.16 Clonogenic assay

A suspension of 4T1 cells, at a concentration of 800 cells per well, was added to a six-well plate and left to incubate for 24 h. They were then subjected to various treatment conditions and cultured for 7 days to allow for the formation of large colonies, defined as containing 50 cells or more. After the colonies had formed, they were treated with fixation and then stained with a 0.5 % crystal violet solution for 10 min to make the colonies visible. Following staining, the plates were photographed, and the number of resulting colonies was counted to assess the clonogenic survival of the cells under different experimental conditions. This assay provided insights into the long-term effects of the treatments on the cells' ability to proliferate and form colonies.

### 2.17 Immunofluorescence staining

4T1 cells, plated in confocal culture dishes, were divided into different groups and exposed to various treatment regimens. Following treatment, all groups were incubated with an immunostaining closure solution at room temperature for 4 h to permeabilize the cells. This was succeeded by a 15-min incubation, followed by an overnight incubation with a mouse anti-γ-H2AX antibody at 4 °C to allow for the specific binding to DNA damage sites. After thorough washing with PBS to remove unbound antibodies, a secondary anti-rabbit Alexa Fluor 488 conjugate was added and incubated for 1 h at room temperature to reveal the locations of the primary antibody. The nuclei were counterstained with DAPI to provide a reference for cellular localization. Ultimately, the extent of DNA damage was analyzed using CLSM, which enabled the visualization and quantification of γ-H2AX foci, serving as a biomarker for double-strand breaks in DNA.

### 2.18 In vitro apoptosis

4T1 cells, at a concentration of 1 × 10^5^ cells per well, were plated into a 6-well plate and incubated for 24 h. Following treatment under various conditions, the cells were further incubated for an additional 24 h and then collected via centrifugation. Afterward, the cells were washed twice with pre-cooled PBS to remove any debris, and re-suspended in 0.1 mL of a 1 × binding buffer solution. Subsequently, the cells were treated with 5 μL of Annexin V-FITC and 5 μL of PI for 10 min to identify apoptotic cells. The stained cells were subjected to flow cytometry analysis to quantify apoptosis, offering a numerical evaluation of cells at various stages of apoptosis and necrosis as indicated by their Annexin V-FITC and PI staining patterns.

### 2.19 Antitumor performance *in vivo*

Tumor growth in BALB/c mice following 4T1 cell inoculation was continuously monitored until the tumors reached approximately 100 mm^3^ in size. At this juncture, all tumor-bearing mice were randomly allocated into five groups (n = 5) with the following treatment regimens: (1) PBS, (2) RT (4 Gy), (3) Ag/Fe-HfO_2_-PEG-RGD, (4) Ag/Fe-HfO_2_-PEG-RGD combined with RT, and (5) Ag/Fe-HfO_2_-PEG-RGD combined with RT and PTT. On days 1, 5, and 9, the experimental mice received intravenous injections of the respective agents (at a dosage of 10 mg/kg) via the tail vein, followed by RT or PTT 6 h post-injection. Throughout the 14-day treatment, the size of the tumors and the body weight of the mice were monitored and recorded. At the end of the treatment period, after the mice were humanely sacrificed, tumor tissues and major organs (including the heart, liver, spleen, lungs, and kidneys) were collected. Subsequently, pathological examination of these tissue sections was conducted using histological staining with various dyes to assess tissue morphology and protein expression, providing insights into the therapeutic efficacy and potential side effects of the treatments.

### 2.20 Statistical analysis

All data were shown as means ± standard deviation (SD). Statistical analysis was assessed using two-way ANOVA.

## 3 Results and discussion

### 3.1 Preparation and characterization of Ag/Fe-HfO_2_-PEG-RGD

**Fig. 1:**
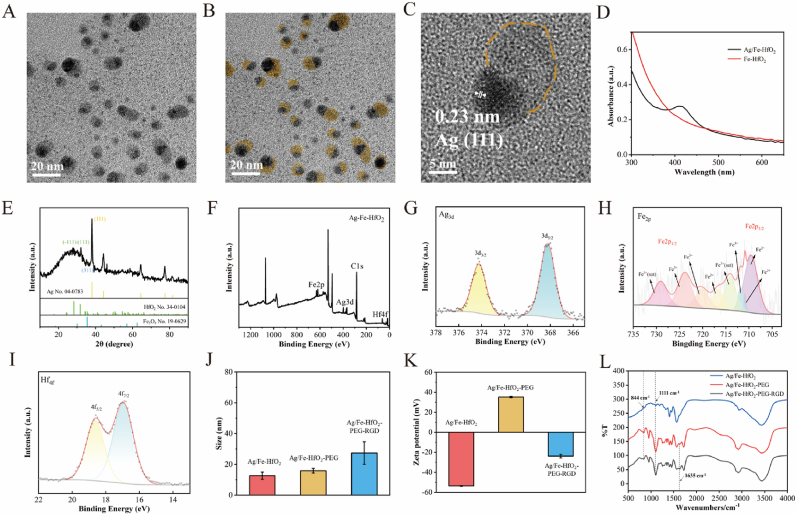
Synthesis and characterization. TEM (A), pseudo-color image of TEM (B), and HRTEM (C) for Ag/Fe-HfO_2_ NPs. (D) UV–vis spectra of Ag/Fe-HfO_2_ and Fe-HfO_2_ NPs solutions. (E) XRD patterns of Ag/Fe-HfO_2_ NPs. The standard lines of JCPDS #04–0783, #19–0629, and #34–0104 are given for comparison. (F) XPS spectra of Ag/Fe-HfO_2_ NPs. XPS high-resolution spectra of (G) Ag 3d, (H) Fe 2p, and (I) Hf 4f for Ag/Fe-HfO_2_ NPs. (J) Particle size of various samples prepared in each step (Ag/Fe-HfO_2_, Ag/Fe-HfO_2_-PEG, and Ag/Fe-HfO_2_-PEG-RGD). (K) Zeta potential measurements for various samples were obtained at each synthesis stage. (L) FT-IR spectroscopy data for the different samples were prepared at each stage of synthesis. (For interpretation of the references to color in this figure legend, the reader is referred to the Web version of this article.)

Fig. 1A presents the TEM image of the Janus NPs, with Fig. 1B showcasing its color-changing property known as pseudo-color image. The Janus structure, characterized by a diameter of roughly 10 nm, is prominently displayed in two distinct contrasting shades within the Janus Ag/Fe-HfO_2_ NPs. The orange color delineates the Fe-HfO_2_ regions. Moreover, the lattice fringe patterns observed in Fig. 1C, with spacings of 0.23 nm, are attributed to the hexagonal arrangement of Ag atoms. Furthermore, we scrutinized the UV–vis–NIR absorption spectra of Fe-HfO_2_ and Janus Ag/Fe-HfO_2_ NPs to confirm the presence of Ag NPs, evidenced by the enhanced absorption at a wavelength of 420 nm, as depicted in Fig. 1D [22,26]. Our findings indicate that there is a direct correlation between the absorption intensity and the concentration of the Janus Ag/Fe-HfO_2_ NPs, as shown in Fig. S1. The structural analysis of Janus Ag/Fe-HfO_2_ NPs was conducted using powder XRD, as illustrated in Fig. 1E. The results proved the formation of HfO_2_ (PDF no. 34–0104), Fe_3_O_4_ (PDF no. 19–0629) and Ag (PDF no. 04–0783). Moreover, the elemental composition of Janus Ag/Fe-HfO_2_ NPs was meticulously examined through XPS, as presented in Fig. 1F. The XPS analysis successfully confirmed the presence of Ag, Fe, and Hf elements within the material [18]. In the high-resolution XPS analysis of the Ag 3d region, as shown in Fig. 1G, the binding energies of 374.2 eV and 368.2 eV are attributed to the Ag^0^ 3d_3/2_ and Ag^0^ 3d_5/2_ peaks, respectively [27]. The deconvolution of the high-resolution Fe 2p spectrum revealed six distinct peaks at 709.02 eV (assigned to Fe^2+^ in octahedral sites), 710.42 eV (attributed to Fe^3+^ in octahedral sites), 712.92 eV (indicative of Fe^3+^ in tetrahedral sites), 719.02 eV (corresponding to Fe^2+^), 723.02 eV (associated with Fe^3+^ in octahedral sites), and 725.87 eV (representing Fe^3+^ in tetrahedral sites), respectively (Fig. 1H). This indicates that both divalent Fe^2+^ and trivalent Fe^3+^ ions coexist within the Janus Ag/Fe-HfO_2_ NPs structure, which is advantageous for its catalase-like (CAT-like) activity. Furthermore, the high-resolution Hf 4f spectrum was deconvoluted into two significant peaks corresponding to the Hf 4f_7/2_ at 15.97 eV and Hf 4f_5/2_ at 17.67 eV, suggesting that hafnium predominantly exists in the form of Hf^4+^ ions, as depicted in Fig. 1I [2]. To enhance biocompatibility and targeting capabilities, the surface of Janus Ag/Fe-HfO_2_ NPs was modified with PEG and RGD. As illustrated in Fig. 1J, the hydrodynamic sizes of the modified nanoparticles were observed to increase; specifically, Ag/Fe-HfO_2_-PEG exhibited a size of 15.9 nm, and Ag/Fe-HfO_2_-PEG-RGD showed a size of 27.4 nm, in comparison to the unmodified Ag/Fe-HfO_2_ at 12.6 nm. Furthermore, the zeta potential of the pristine Ag/Fe-HfO_2_ was measured at −53.62 mV (Fig. 1K). This value changed to 35.24 mV upon PEG modification, which can be attributed to the interaction between the NH_2_ groups of PEG and the COOH groups of PAA on the surface, thereby reducing the overall negative charge. The addition of RGD further altered the zeta potential to −23.89 mV for Ag/Fe-HfO_2_-PEG-RGD, a change that is conducive to improved circulation within the bloodstream. As shown in Fig. 1L, in comparison to the unmodified Ag/Fe-HfO_2_, infrared spectroscopy analysis revealed distinctive peaks for the modified nanoparticles. Specifically, Ag/Fe-HfO_2_-PEG exhibited characteristic peaks at 844 cm^−1^ and 1111 cm^−1^, which are indicative of the PEG moiety. Additionally, Ag/Fe-HfO_2_-PEG-RGD displayed a characteristic peak at 1653 cm^−1^, corresponding to the presence of the RGD peptide. These observations confirm the successful functionalization of the Ag/Fe-HfO_2_ surface with PEG and RGD. The stability of Ag/Fe-HfO_2_-PEG-RGD in different media (H_2_O, PBS, 1640, and FBS) was verified (Fig. S2), which is beneficial for further research *in vitro* and *in vivo*.

### 3.2 Photothermal properties, MRI ability, and enzymatic catalytic activity of the Janus Ag/Fe-HfO_2_ NPs

**Fig. 2:**
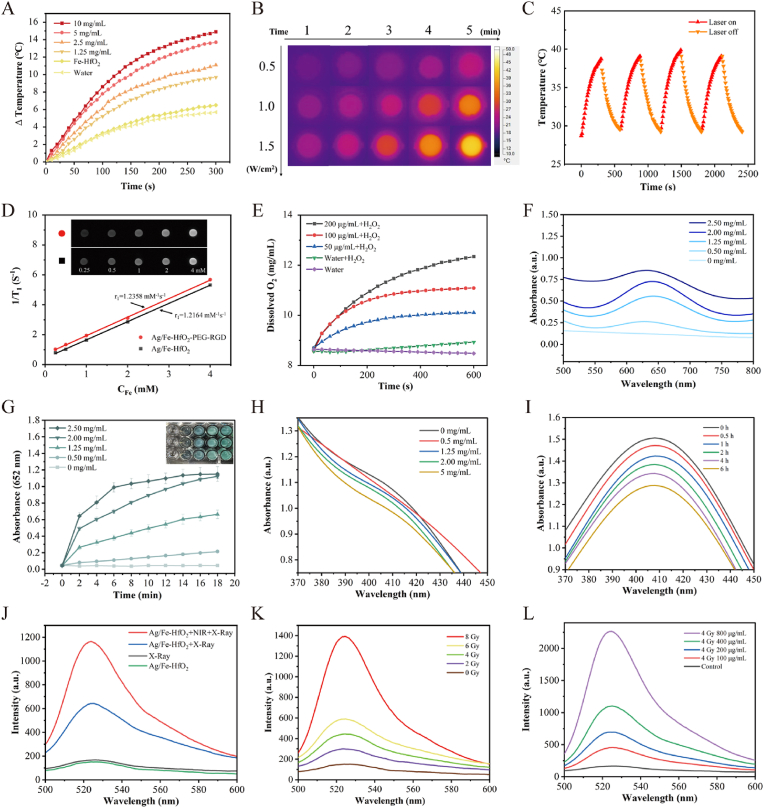
Photothermal properties, MRI ability, and enzymatic catalytic activity of the Janus Ag/Fe-HfO_2_ NPs. (A) Temperature elevation of water, Fe-HfO_2_, and Janus Ag/Fe-HfO_2_ NPs solution with various concentrations. (B) *In vitro* photothermal images of Janus Ag/Fe-HfO_2_ NPs solution at various power upon 808 nm laser irradiation. (C) Photothermal stability of Janus Ag/Fe-HfO_2_ NPs upon 808 nm laser treatment for four cycles. (D) Relaxation rate and magnetic resonance imaging of Janus Ag/Fe-HfO_2_ NPs and Ag/Fe-HfO_2_-PEG-RGD. (E) O_2_ generation in the solution containing H_2_O_2_ in the presence of Janus Ag/Fe-HfO_2_ NPs with different concentrations (200, 100, 50, and 0 mg/mL). (F) UV spectra of TMB reaction by Ag/Fe-HfO_2_ NPs at 652 nm, was measured with varying concentrations (0, 0.50, 1.25, 2.00, and 2.50 mg/mL). (G) The absorbance over time, resulting from the catalytic oxidation of TMB by Ag/Fe-HfO_2_ NPs at 652 nm, was measured with varying concentrations (0, 0.50, 1.25, 2.00, and 2.50 mg/mL). Inset picture: A photo for observing the color of different samples. (H) GSH depletion by Janus Ag/Fe-HfO_2_ NPs with various concentrations (0, 0.5, 1.25, 2.00, and 5.00 mg/mL). (I) GSH depletion by Janus Ag/Fe-HfO_2_ NPs (5 mg/mL) with different reaction times. (J) Evaluation of ROS generation ability of Janus Ag/Fe-HfO_2_ NPs with various treatments. Evaluation of ROS generation ability of Janus Ag/Fe-HfO_2_ NPs upon different radiation doses (K) and with different concentrations upon 4 Gy (L). (For interpretation of the references to color in this figure legend, the reader is referred to the Web version of this article.)

The study investigated the photothermal characteristics of Janus Ag/Fe-HfO_2_ NPs by observing the temperature changes in Janus Ag/Fe-HfO_2_ NPs samples when exposed to an 808 nm laser with a power density of 1.0 W/cm^2^, as shown in Fig. 2A. The nanocomposites displayed a pronounced hyperthermic response that was dependent on concentration, with the temperature increase becoming more significant as the concentration of the sample increased. At a 10 mg/mL concentration, the solution temperature rose sharply by 14.9 °C within 5 min, while the control group (water and Fe-HfO_2_) showed a lower temperature rise. Meanwhile, the temperature of the same concentration of solutions increases with the augmented power of laser irradiation (Fig. S3). Fig. 2B illustrates the temperature variations of Janus Ag/Fe-HfO_2_ NPs samples with varying laser power (0.5, 1.0, and 1.5 W/cm^2^) after a 5-min laser irradiation, as captured by a thermal imaging camera. Additionally, the photothermal performance of Janus Ag/Fe-HfO_2_ NPs remained largely unchanged after undergoing four cycles of heating and cooling (as depicted in Fig. 2C), which attests to the excellent stability of Janus Ag/Fe-HfO_2_ NPs, making it a reliable long-term photothermal agent. Photothermal conversion efficiency is an important indicator for measuring the photothermal performance of nanomaterials. The τ values of Ag/Fe-HfO_2_ NPs were calculated to be 241.9, and the calculated photothermal conversion efficiencies of Ag/Fe-HfO_2_ NPs at an 808 nm laser irradiation (1.0 W/cm^2^) were 17.0 % (Fig. S4). This indicates that Janus Ag/Fe-HfO_2_ NPs have a unique ability to convert NIR light into heat [22,28]. To assess the MRI capabilities, we determined the *T*_*1*_ relaxation times of Janus Ag/Fe-HfO_2_ NPs and Ag/Fe-HfO_2_-PEG-RGD NPs in deionized (DI) water at different iron concentrations using a 0.5-T MRI scanner (as shown in Fig. 2D). To calculate the r_1_ relaxivity, a linear regression analysis was conducted with 1/*T*_*1*_ plotted against Fe concentration, yielding an r_1_ relaxivity value of 1.22 mM^−1^ s^−1^ for Janus Ag/Fe-HfO_2_ NPs from the gradient of the fitted line [29]. After the surface is modified with PEG and RGD, the r_1_ relaxivity value of Ag/Fe-HfO_2_-PEG-RGD is 1.24 mM^−1^ s^−1^. The *T*_*1*_-weighted MR images of nanoparticles in aqueous dispersions demonstrate a correlation between increasing signal intensity and elevated iron concentrations. This correlation suggests the potential of these nanoparticles for use in *T*_*1*_-weighted MRI applications [30]. The catalase-like activity of Janus Ag/Fe-HfO_2_ NPs was demonstrated by a rapid increase in dissolved oxygen upon its introduction to an H_2_O_2_ solution, a more pronounced effect than observed with water or water plus H_2_O_2_ without the catalyst [17]. Following a 10-min reaction, the dissolved O_2_ concentrations in the solutions with 200, 100, 50, and 0 μg/mL of Janus Ag/Fe-HfO_2_ NPs increased to 12.34, 11.09, 10.11, and 8.93 mg/mL, respectively (Fig. 2E). This finding demonstrates the superior capability of Janus Ag/Fe-HfO_2_ NPs to substantially boost oxygen generation in biological systems. Fig. 2F shows that the absorbance at 652 nm increases with increasing concentration. Fig. 2G illustrates the absorbance of the reaction mixture at 652 nm over time, highlighting the linear segment of the curve obtained. The gradual decrease in the absorbance of MB with the progression of the reaction time indicates that the Janus Ag/Fe-HfO_2_ NPs are capable of catalyzing H_2_O_2_ to generate hydroxyl radicals, which are lethal to tumor cells. This finding underscores the potential of these nanocomposites as a catalyst in therapeutic applications [28]. ESR spectroscopy using a 5,5-dimethyl-1-pyrroline N-oxide (DMPO) probe further validate this result, as the characteristic (1:2:2:1) ·OH signal is observed in Janus Ag/Fe-HfO_2_ NPs (Fig. S5). In the coexistence of Ag/Fe-HfO_2_ and H_2_O_2_, the 652 nm absorbance of TMB was recorded for the calculation of kinetic parameters (Fig. S6). The Lineweaver-Burk plot is obtained by the Lambert-Beer law and the Michaelis-Menten equation, and the Michaelis constant (K_m_) and the maximum reaction speed (V_max_) for Ag/Fe-HfO_2_ are estimated to be 1.05 mM and 6.67 × 10^−8^ M s^−1^, respectively. It is noteworthy that the efficacy of current CDT and RT is often hindered by the antioxidant defense mechanisms within tumor tissues. This is primarily due to the high levels of GSH molecules that neutralize the ROS generated, such as ·OH and singlet oxygen (^1^O_2_) [31]. However, the Janus Ag/Fe-HfO_2_ NPs not only catalyze the production of these species but also emulate the function of glutathione oxidase (GSHOD), thereby depleting GSH levels. This hypothesis is corroborated by the observation that the absorbance of 5,5′-dithiobis-(2-nitrobenzoic acid), a GSH probe, diminishes in a manner that is dependent on the concentration of the Janus Ag/Fe-HfO_2_ NPs (Fig. 2H) [32]. In addition, with the increase in reaction time, the absorbance at 410 nm decreased (Fig. 2I). The fluorescence emission spectra of DCFH in the sample solutions are depicted in Fig. 2J [33]. In the absence of 808 nm laser irradiation, Janus Ag/Fe-HfO_2_ NPs still exhibited a significantly higher enhancement in ROS generation compared to both water and Janus Ag/Fe-HfO_2_ NPs alone after X-ray exposure. When subjected to X-ray radiation, Janus Ag/Fe-HfO_2_ NPs under 808 nm laser irradiation led to a markedly higher production of ROS compared to the scenario without 808 nm laser irradiation. This observation underscores the pronounced photothermal-enhanced radiosensitizing effect of Janus Ag/Fe-HfO_2_ NPs. Fig. 2K and L illustrate that as the concentration of Janus Ag/Fe-HfO_2_ NPs and the X-ray dosage increase, the production of ROS correspondingly intensifies. This finding suggests that the high-Z element Hf also plays a pivotal role in the radiosensitizing action of Janus Ag/Fe-HfO_2_ NPs.

### 3.3 In vitro anticancer performance of Ag/Fe-HfO_2_-PEG-RGD

**Fig. 3:**
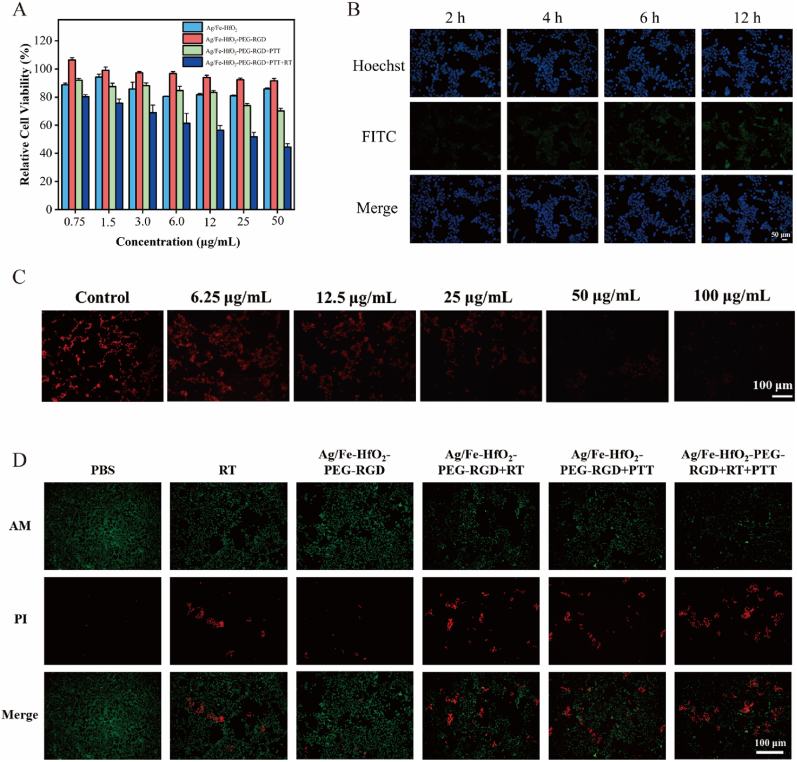
*In vitro* anticancer performance of Ag/Fe-HfO_2_-PEG-RGD. (A) Relative cell viability of 4T1 cells after cultivated with Janus Ag/Fe-HfO_2_ NPs, Ag/Fe-HfO_2_-PEG-RGD, Ag/Fe-HfO_2_-PEG-RGD + PTT, and Ag/Fe-HfO_2_-PEG-RGD + PTT + RT. Data were given as mean ± S.D. (n = 3). (B) Cellular uptake of 4T1 cells incubated with FITC-labeled Ag/Fe-HfO_2_-PEG-RGD for 2, 4, 6, and 12 h. Scale bar = 50 μm. (C) [Ru(dpp)_3_] Cl_2_ probe staining for detecting the change of O_2_ in 4T1 cells after indicated treatments. Scale bar = 100 μm. (D) Confocal microscopy images of 4T1 tumor cells were obtained after staining with Calcein-AM (which exhibits green fluorescence in viable cells) and PI (which shows red fluorescence in dead cells), following the specified treatments. Scale bar = 100 μm. (For interpretation of the references to color in this figure legend, the reader is referred to the Web version of this article.)

To delve deeper into the study of PTT, RT, and nanozyme catalytic activities, Ag/Fe-HfO_2_-PEG-RGD nanocomposites exhibit remarkable antitumor efficacy *in vitro*. This discovery underscores the potential of these nanocomposites in cancer therapy. Initially, the *in vitro* cell viability of 4T1 cells was assessed using standard MTT assays. Fig. 3A depicts the relative cell viability of 4T1 cells after 24-h exposure to varying concentrations of Ag/Fe-HfO_2_, Ag/Fe-HfO_2_-PEG-RGD, Ag/Fe-HfO_2_-PEG-RGD + PTT and Ag/Fe-HfO_2_-PEG-RGD + PTT + RT. At concentrations up to 50 μg/mL, Ag/Fe-HfO_2_-PEG-RGD nanocomposites demonstrated minimal cytotoxicity, with 4T1 cell viability remaining as high as 91.66 %. However, upon exposure to 808 nm laser irradiation for 5 min, the cell viability dropped to 70.08 % at a concentration of 50 μg/mL Ag/Fe-HfO_2_-PEG-RGD. Following combined radiotherapy, the cell survival rate further decreased to 43.13 %, indicating a significant PTT and RT effect. Meanwhile, to evaluate the cytotoxic effects of Ag/Fe-HfO_2_-PEG-RGD on normal cells, we conducted an MTT assay using HC11 cells. The results demonstrated that cell viability remained above 80 % even at the concentration of 100 μg/mL (Fig. S7). These findings collectively confirm the favorable biocompatibility and safety profile of the material *in vitro*. FITC-tagged Janus Ag/Fe-HfO_2_ NPs and Ag/Fe-HfO_2_-PEG-RGD were introduced to 4T1 cells, each at different intervals. Cellular fluorescence was measured after incubation periods of 2, 4, 6, and 12 h. As depicted in Fig. 3B and S8, the fluorescence intensity for both sets of NPs escalated alongside the extension of incubation time. Specifically, 4T1 cells treated with FITC-tagged Ag/Fe-HfO_2_-PEG-RGD displayed more intense fluorescence compared to those treated with FITC-tagged Ag/Fe-HfO_2_ at the same time points. The RGD modification on the surface of Ag/Fe-HfO_2_-PEG-RGD enhances its targeting capability, resulting in a stronger interaction with 4T1 cells [34,35]. To assess the CAT-like activity of Ag/Fe-HfO_2_-PEG-RGD, the [Ru(dpp)_3_]Cl_2_ probe was employed as an oxygen sensor, which is quenched in the presence of O_2_ [36]. Cells treated with various concentrations of Ag/Fe-HfO_2_-PEG-RGD showed a reduction in red fluorescence compared to the PBS control group. With increasing concentrations, the red fluorescence intensity diminished, nearly disappearing at a concentration of 100 μg/mL Ag/Fe-HfO_2_-PEG-RGD, suggesting that these nanocomposites possess the capacity to reduce tumor hypoxia (Fig. 3C) [37]. To visualize the therapy ability, AM/PI dual stains were employed to indicate the viable and dead cells upon various treatments [38,39]. In line with the cytotoxicity findings, only a small number of cells succumbed after exposure to Ag/Fe-HfO_2_-PEG-RGD. Compared with the RT (X-ray, 4 Gy) or Ag/Fe-HfO_2_-PEG-RGD groups alone, a noticeably higher number of dead cells were observed in the Ag/Fe-HfO_2_-PEG-RGD + RT and Ag/Fe-HfO_2_-PEG-RGD + PTT groups, suggesting an augmented response to RT and PTT. Moreover, the combined PTT and RT of Ag/Fe-HfO_2_-PEG-RGD nearly eradicated the tumor cells, highlighting the potent synergistic therapeutic effect (Fig. 3D).

**Fig. 4:**
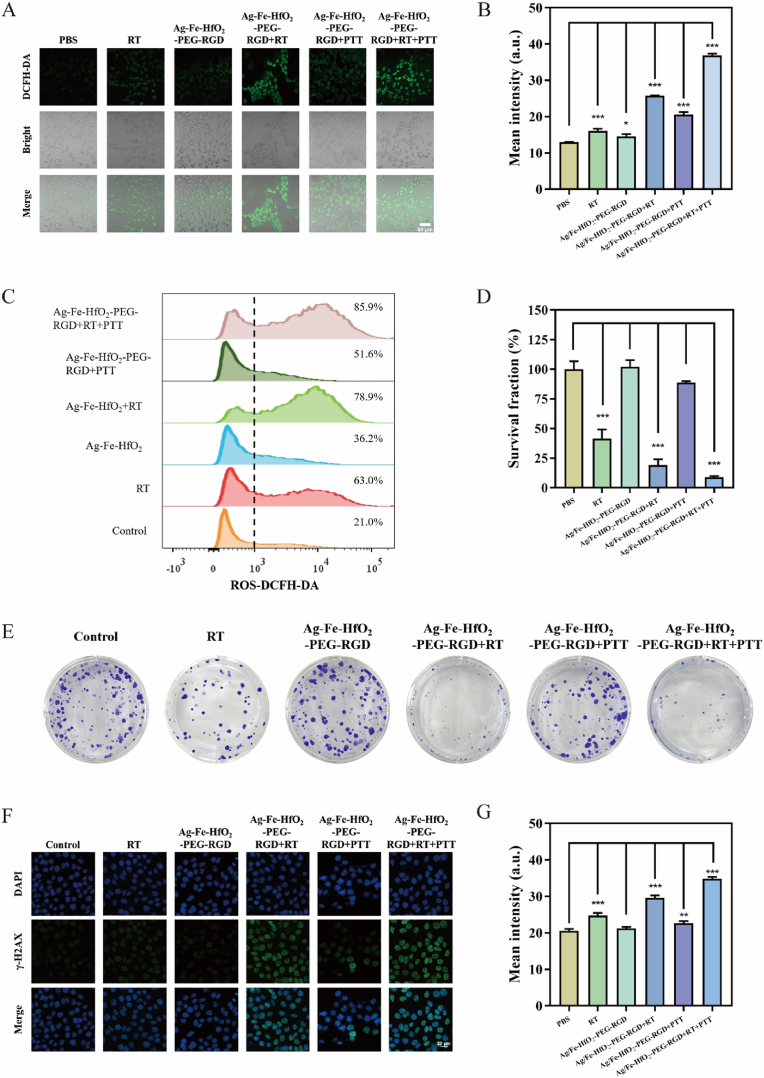
The radiosensitize effect of Ag/Fe-HfO_2_-PEG-RGD. (A) Fluorescence images and (B) mean fluorescence intensity of 4T1 cells cultivated with different treatments using DCFH-DA as the ROS probe. Scale bar = 60 μm. (C) Flow cytometric investigations of intracellular ROS levels of 4T1 tumor cells after corresponding treatments. (D and E) The effect of different treatments on the formation of 4T1 cell colonies was assessed by crystal violet staining. (F) Confocal images and (G) mean fluorescence intensity of γ-H2AX-stained 4T1 cells after different treatments. Scale bar = 20 μm. Data were given as mean ± S.D. (n = 3). (For interpretation of the references to color in this figure legend, the reader is referred to the Web version of this article.)

The radiosensitizing effect of Ag/Fe-HfO_2_-PEG-RGD was evaluated by assessing intracellular ROS production and DNA damage. Intracellular ROS levels were visualized using the DCFH-DA probe. This method provides a clear indication of the ability to enhance the effectiveness of RT. Cells treated with PBS, RT, or Ag/Fe-HfO_2_-PEG-RGD alone displayed only faint green fluorescence. In contrast, the groups treated with Ag/Fe-HfO_2_-PEG-RGD + RT (mean intensity: 25.76), Ag/Fe-HfO_2_-PEG-RGD + PTT (mean intensity: 20.57), and Ag/Fe-HfO_2_-PEG-RGD + RT + PTT (mean intensity: 36.90) exhibited significantly stronger fluorescence. This indicates a marked increase in ROS production when Ag/Fe-HfO_2_-PEG-RGD is used in conjunction with X-ray exposure or PTT (Fig. 4A and B). The flow cytometric analysis data further corroborated these results (Fig. 4C). A clonogenic assay was conducted to further assess the combined inhibitory impact of Ag/Fe-HfO_2_-PEG-RGD-mediated PTT and RT on 4T1 tumor cells. The results, as depicted in Fig. 4D and E, indicate that nearly all cells were eradicated following sequential exposure to 808 nm laser irradiation and X-ray radiation, with the cell survival rate dropping to approximately 8.83 %. However, when treated with Ag/Fe-HfO_2_-PEG-RGD alone, Ag/Fe-HfO_2_-PEG-RGD + RT or Ag/Fe-HfO_2_-PEG-RGD + PTT, the survival rates were 102.17 %, 19.15 % or 88.83 %, respectively. These findings suggest that Ag/Fe-HfO_2_-PEG-RGD facilitates the synergistic therapeutic effects of PTT and RT. Furthermore, the cell-killing capability of Ag/Fe-HfO_2_-PEG-RGD in conjunction with X-ray radiation was significantly more potent than X-ray radiation alone, with survival rates of 19.15 % and 41.51 % after their respective treatments. These outcomes collectively suggest that Ag/Fe-HfO_2_-PEG-RGD holds promise as an effective sensitizer for RT. The phosphorylation of histone H2AX, known as γ-H2AX, was utilized to further investigate DNA damage, acting as an indicator for DNA double-strand breaks caused by radiation. The findings indicated that cells treated with Ag/Fe-HfO_2_-PEG-RGD + RT + PTT (mean intensity: 34.89) exhibited a notably higher presence of γ-H2AX-positive foci within the nucleus than those treated with RT alone (mean intensity: 24.75), Ag/Fe-HfO_2_-PEG-RGD alone (mean intensity: 21.22), Ag/Fe-HfO_2_-PEG-RGD + RT (mean intensity: 29.56), or Ag/Fe-HfO_2_-PEG-RGD + PTT (mean intensity: 22.69) (Fig. 4F and G). Using Annexin V-FITC/PI double staining flow cytometry to assess apoptosis in each experimental group (Fig. S9), it was found that the percentage of apoptotic cells (the sum of Q2 and Q3 values) significantly increased after treatment with Ag/Fe-HfO_2_-PEG-RGD + RT + PTT. Consequently, the findings collectively demonstrated that the combination of Ag/Fe-HfO_2_-PEG-RGD with RT and PTT yielded superior tumor treatment outcomes compared to other groups. This suggests that the elevated temperature and the oxygen released by Ag/Fe-HfO_2_-PEG-RGD significantly enhance the effectiveness of RT [40].

### 3.4 In vivo anticancer performance of Ag/Fe-HfO_2_-PEG-RGD

**Fig. 5:**
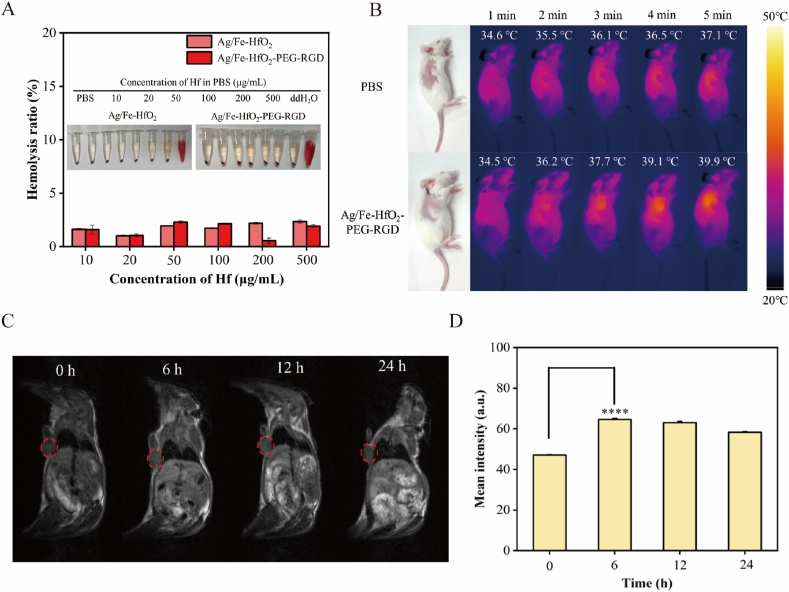
Hemolysis analysis, infrared thermal imaging, and MRI of Ag/Fe-HfO_2_-PEG-RGD NPs. (A) Hemolysis analysis of Ag/Fe-HfO_2_ NPs and Ag/Fe-HfO_2_-PEG-RGD. (B) Infrared thermal imaging captured the temperature profiles at the tumor sites treated with Ag/Fe-HfO_2_-PEG-RGD in comparison to the control group treated with PBS. (C) *In vivo T*_*1*_-weighted MRI of Ag/Fe-HfO_2_-PEG-RGD at different post-injection times. (D) The mean intensity of the *T*_*1*_-weighted MRI of the tumor.

To determine the suitability of Ag/Fe-HfO_2_-PEG-RGD for intravenous administration, a preliminary hemolysis investigation was conducted [41]. The outcomes indicated that neither Ag/Fe-HfO_2_ nor Ag/Fe-HfO_2_-PEG-RGD triggered hemolysis at various concentrations (Fig. 5A). The application of bioimaging technology ensures that laser treatment is accurately targeted at the tumor site [42,43]. We monitored the *in vivo* photothermal effects of Ag/Fe-HfO_2_-PEG-RGD nanocomposites at designated irradiation intervals using infrared thermal imaging, as depicted in Fig. 5B. In contrast to the PBS group, when the mouse tumor site was irradiated with 808 nm light, a significant increase in temperature was noted in the Ag/Fe-HfO_2_-PEG-RGD group. Within just 5 min, the tumor temperature escalated to 39.9 °C (Fig. S10). As shown in Fig. 5C and D, the *in vivo T*_*1*_-weighted MRI demonstrated peak signal intensity at the 6-h mark following intravenous administration, which was then followed by a gradual decline in signal strength. Therefore, the Ag/Fe-HfO_2_-PEG-RGD nanocomposites can effectively act as a nanotherapeutic agent for MRI imaging-guided synergistic tumor treatment.

**Fig. 6:**
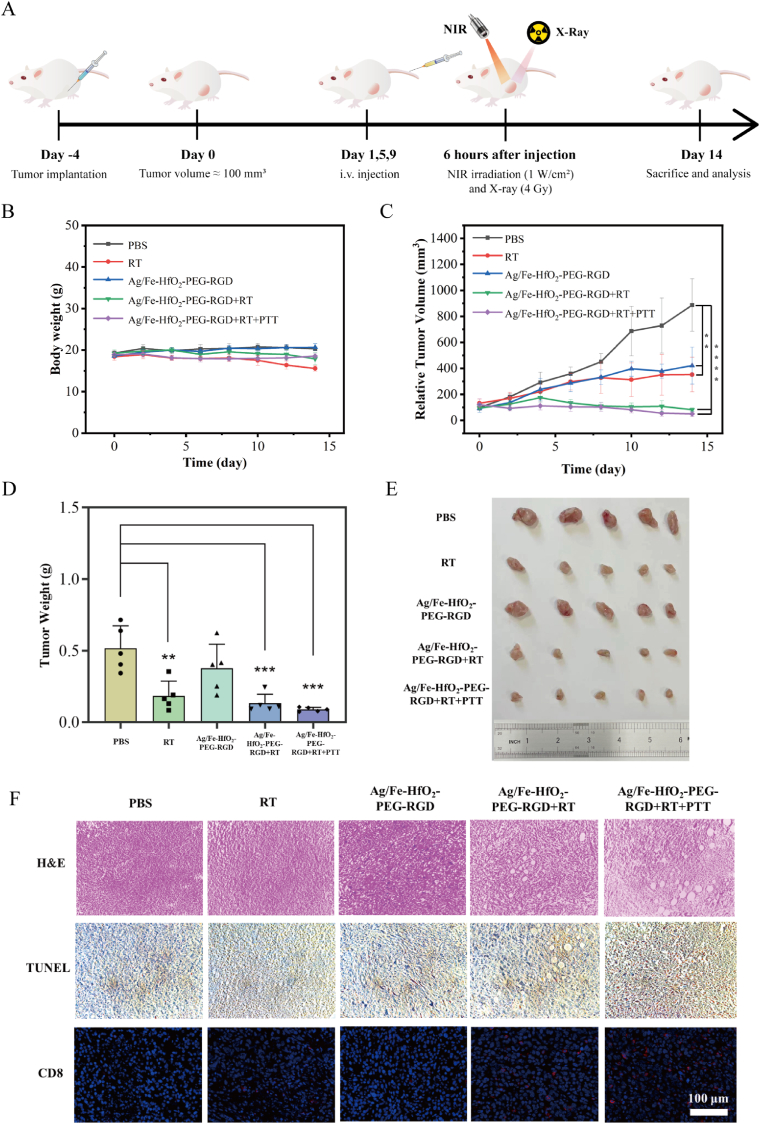
*In vivo* Anticancer Efficacy of Ag/Fe-HfO_2_-PEG-RGD. (A) Schematic representation of *in vivo* experimental protocol. (B) Body weight and (C) relative tumor volumes of mice in various treatment groups (n = 5). (D) Tumor weights and representative photographs (E) of excised tumors on day 14 following treatment with different groups. (F) H&E, TUNEL, and CD8 staining of tumor sections from various treatment groups after 14 days. Scale bar = 100 μm.

4T1 subcutaneous tumor model in BALB/c mice was developed to assess the antitumor effect elicited by the combination of Ag/Fe-HfO_2_-PEG-RGD with RT and PTT. As depicted in Fig. 6A, 4T1 cells were injected subcutaneously into the left flank of the mice four days after the initial inoculation of tumors on the right side. Subsequently, these five distinct treatment groups were treated selectively with intravenous (i.v.) injections of either PBS or Ag/Fe-HfO_2_-PEG-RGD, X-ray irradiation, or NIR targeted at the primary tumors at specified time points. Throughout the treatment period, there were no significant alterations in body weight observed across all groups (Fig. 6B). As depicted in Fig. 6C, from the day of administration, comparable to the tumor volume in the PBS group, the Ag/Fe-HfO_2_-PEG-RGD group demonstrated a noticeable inhibitory effect on tumor growth. The Ag/Fe-HfO_2_-PEG-RGD Janus nanoparticles were capable of inducing the production of ROS in tumor tissues and effectively executed a CDT effect, yet this was not sufficient to completely eradicate the tumors. The tumor sizes in both the RT group and the Ag/Fe-HfO_2_-PEG-RGD + RT group were significantly reduced, highlighting the potent inhibitory effect of RT on tumor growth and the superior radiotherapy sensitization of Ag/Fe-HfO_2_-PEG-RGD. Notably, the tumors in the Ag/Fe-HfO_2_-PEG-RGD + RT + PTT group nearly vanished, outperforming the results observed in the Ag/Fe-HfO_2_-PEG-RGD + RT group. These comparative results underscored the fact that the combination of RT and PTT was more efficacious in suppressing tumor growth, achieving a tumor inhibition rate as high as 94.38 %. Combined with the results of Fig. 6D and E, this synergistic therapeutic approach offered a promising strategy for enhancing the efficacy of cancer treatments. H&E and TUNEL staining of tumor sections revealed that the Ag/Fe-HfO_2_-PEG-RGD + RT + PTT group caused the most significant tumor damage due to extensive cell necrosis and apoptosis, as shown in Fig. 6F. Immunofluorescence examination of tumor tissue sections demonstrated that either RT or Ag/Fe-HfO_2_-PEG-RGD alone resulted in a negligible infiltration of CD8^+^ T cells within the tumor tissue. In contrast, the combined treatment with Ag/Fe-HfO_2_-PEG-RGD + RT + PTT led to a significant increase in CD8^+^ T cell infiltration compared to the Ag/Fe-HfO_2_-PEG-RGD + RT group, suggesting enhanced anti-tumor immune response. The findings indicate that the integration of Ag/Fe-HfO_2_-PEG-RGD with RT and PTT has the potential to boost the infiltration of CD8^+^ T cells and amplify the tumor-fighting capabilities of the immune system.

## 4 Conclusions

In summary, we synthesized a novel Janus Ag/Fe-HfO_2_ NPs in a single step. This NP serves as a versatile nanoradiosensitizer with photothermal capabilities, enzyme-mimicking properties, and MRI capabilities. Its surface was modified with PEG to enhance biocompatibility and functionalized with RGD targeting peptides that specifically bind to tumor cells. This advancement effectively addresses the primary drawbacks of conventional RT by consolidating multiple functionalities within a unified system. *T*_*1*_-weighted MRI-guided RT, along with enzymatic activity to combat hypoxia, produce hydroxyl radicals, deplete GSH levels, and photothermal heating, works synergistically to enhance the therapeutic impact of RT. Concurrently, it triggers tumor-specific immune responses, resulting in an increased infiltration of CD8^+^ cells within the tumor tissue. Considering the clinical use of HfO_2_ (commercially known as NBTXR3 or Henisfy), this integrated multimodal nanotherapeutic platform, which capitalizes on the potent augmentation of RT effects, offers a promising strategy in the fight against cancer.

## CRediT authorship contribution statement

**Xinying Liu:** Writing – original draft, Methodology, Investigation, Data curation, Conceptualization. **Baohui Liu:** Investigation. **Conglong Chen:** Investigation. **Chaowei Hong:** Validation, Investigation. **Jia Xu:** Investigation. **Yixuan Ruan:** Investigation. **Ling Huang:** Investigation. **Shanni Hong:** Supervision, Funding acquisition. **Wei Chen:** Writing – review & editing, Supervision, Resources, Conceptualization. **Ye Kuang:** Writing – review & editing, Supervision, Resources, Funding acquisition.

## Declaration of competing interest

The authors declare that they have no known competing financial interests or personal relationships that could have appeared to influence the work reported in this paper.

## Data Availability

Data will be made available on request.

## References

[1] Wang J., Pan J., Tang Y., Chen J., Fei X., Xue W., Liu X. (2023). Advances of hafnium based nanomaterials for cancer theranostics. Front. Chem..

[2] Cao Y., Ding S., Hu Y., Zeng L., Zhou J., Lin L., Zhang X., Ma Q., Cai R., Zhang Y., Duan G., Bian X., Tian G. (2024). An immunocompetent hafnium oxide-based sting nanoagonist for cancer radio-immunotherapy. ACS Nano.

[3] Ding S., Chen L., Liao J., Huo Q., Wang Q., Tian G., Yin W. (2023). Harnessing hafnium‐based nanomaterials for cancer diagnosis and therapy. Small.

[4] Nguyen T., Shin S., Choi H., Bark C. (2022). Engineered cancer cell membranes: an emerging agent for efficient cancer theranostics. Exploration.

[5] Zhou L., Huang Y., Wu Y., Tang S. (2024). Nanoparticle targeting cGAS-STING signaling in disease therapy. Nano Res..

[6] Wang Z., Chen F., Cao Y., Zhang F., Sun L., Yang C., Xie X., Wu Z., Sun M., Ma F., Shao D., Leong K., Pei R. (2024). An engineered nanoplatform with tropism toward irradiated glioblastoma augments its radioimmunotherapy efficacy. Adv. Mater..

[7] Sun L., Cao Y., Lu Z., Ding P., Wang Z., Ma F., Wang Z., Pei R. (2022). A hypoxia-irrelevant Fe-doped multivalent manganese oxide sonosensitizer a vacancy engineering strategy for enhanced sonodynamic therapy. Nano Today.

[8] Zhou F., Huang L., Li S., Yang W., Chen F., Cai Z., Liu X., Xu W., Lehto V., Lächelt U., Huang R., Shi Y., Lammers T., Tao W., Xu Z., Wagner E., Xu Z., Yu H. (2024). From structural design to delivery: mRNA therapeutics for cancer immunotherapy. Exploration.

[9] Jiang Q., Liu L., Li Q., Cao Y., Chen D., Du Q., Yang X., Huang D., Pei R., Chen X., Huang G. (2021). NIR-laser-triggered gadolinium-doped carbon dots for magnetic resonance imaging, drug delivery and combined photothermal chemotherapy for triple negative breast cancer. J. Nanobiotechnol..

[10] Pei P., Wang Y., Shen W., He Q., Han X., Zhang C., Xie Y., Zhou G., Zhao Y., Hu L., Yang K. (2024). Oxygen‐driven cuproptosis synergizes with radiotherapy to potentiate tumor immunotherapy. Aggregate.

[11] Chen J., Ran P., Xu Y., Khouchani M., Li X., Jian L., Abdelmajid T., Aittahssaint N., Yang Q., Li J., Zhao L. (2025). Biomimetic multifunctional nanoparticles for improved radiotherapy and immunotherapy in cancer treatment, mater. Today Bio.

[12] Zhu L., Chen G., Wang Q., Du J., Wu S., Lu J., Liu B., Miao Y., Li Y. (2024). High-Z elements dominated bismuth-based heterojunction nano-semiconductor for radiotherapy-enhanced sonodynamic breast cancer therapy. J. Colloid Interface Sci..

[13] Sherstiuk A.A., Tsymbal S.A., Fakhardo A.F., Morozov V.N., Krivoshapkina E.F., Hey-Hawkins E., Krivoshapkin P.V. (2021). Hafnium oxide-based nanoplatform for combined chemoradiotherapy. ACS Biomater. Sci. Eng..

[14] Li Y., Qi Y., Zhang H., Xia Z., Xie T., Li W., Zhong D., Zhu H., Zhou M. (2020). Gram-scale synthesis of highly biocompatible and intravenous injectable hafnium oxide nanocrystal with enhanced radiotherapy efficacy for cancer theranostic. Biomaterials.

[15] Zhang C., Li D., Zhang X., Dai R., Kang W., Li Y., Liu Q., Gao M., Zheng Z., Zhang R., Wen Z. (2024). Dual regulation of osteosarcoma hypoxia microenvironment by a bioinspired oxygen nanogenerator for precise single-laser synergistic photodynamic/photothermal/induced antitumor immunity therapy. Mater. Today Bio.

[16] Sajid S., Jahan N., Huma Z., Ishaq A.M., Zada A., Ibrar A., Abbas A.G., Noureen L., Ayaz M., Arain S., Saeed F. (2024). Fabrication, characterization, and in vitro testing of quercetin–copper(ii) complex. Nano Biomed. Eng..

[17] Cao C., Zou H., Yang N., Li H., Cai Y., Song X., Shao J., Chen P., Mou X., Wang W., Dong X. (2021). Fe_3_O_4_/Ag/Bi_2_MoO_6_ photoactivatable nanozyme for self‐replenishing and sustainable cascaded nanocatalytic cancer therapy. Adv. Mater..

[18] Wang Y., Wang D., Zhang Y., Xu H., Shen L., Cheng J., Xu X., Tan H., Chen X., Li J. (2022). Tumor microenvironment-adaptive nanoplatform synergistically enhances cascaded chemodynamic therapy. Bioact. Mater..

[19] Zhang M., Wang J., Gong Y. (2024). Atomically dispersed silver atoms incorporated in spinel cobalt oxide (Co_3_O_4_) for boosting oxygen evolution reaction. J. Colloid Interface Sci..

[20] Ji W., Ji X., Cao L., Wang W., Chen S. (2024). Silver sulfide anchored bismuth molybdate p-n heterojunction nano-coating with excellent photo-thermal self-healing performance. J. Colloid Interface Sci..

[21] Yu T., Wu J., Shen Y., Penkova A., Qi W., Su R. (2024). Transparent coating based on multienzyme-mimicking Janus nanozyme for synergetic biofouling control in seawater. Chem. Eng. J..

[22] Zang P., Du Y., Yu C., Yang D., Gai S., Feng L., Liu S., Yang P., Lin J. (2023). Photothermal-actuated thermoelectric therapy by harnessing janus-structured Ag–Ag_2_S nanoparticles with enhanced antitumor efficacy. Chem. Mater..

[23] Moonshi S.S., Vazquez-Prada K.X., Tang J., Westra van Holthe N.J., Cowin G., Wu Y., Tran H.D.N., McKinnon R., Bulmer A.C., Ta H.T. (2023). Spiky silver-iron oxide nanohybrid for effective dual-imaging and synergistic thermo-chemotherapy. ACS Appl. Mater. Interfaces.

[24] Nie C., Du P., Zhao H., Xie H., Li Y., Yao L., Shi Y., Hu L., Si S., Zhang M., Gu J., Luo L., Sun Z. (2020). Ag@TiO_2_ nanoprisms with highly efficient near-infrared photothermal conversion for melanoma therapy. Chem. Asian J..

[25] Xu M., Qian Y., Li X., Gu B., He S., Lu X., Song S. (2024). Janus ACSP nanoparticle for synergistic chemodynamic therapy and radiosensitization. ACS Appl. Mater. Interfaces.

[26] Wang J., Luo D., Cai Y., Li X., Chen H., Xu J. (2022). A plasmonic Au-Ag janus nanoprobe for monitoring endogenous hydrogen sulfide generation in living cells. Biosens. Bioelectron..

[27] Wang R., Wang J., Wang X., Song G., Ye L., Gu W. (2022). BSA-templated ultrasmall Ag/Gd_2_O_3_ as a self-enabled nanotheranostic for MR/CT/PA tri-modality imaging and photothermal therapy. Biomater. Sci..

[28] Xu M., Zhao R., Liu B., Geng F., Wu X., Zhang F., Shen R., Lin H., Feng L., Yang P. (2024). Ultrasmall copper-based nanoplatforms for NIR-II light-triggered photothermal/photodynamic and amplified nanozyme catalytic therapy of hypoxic tumor. Chem. Eng. J..

[29] Cao Y., Pei R., Lu Z., Yan J., Xu M., Sun L., Liu J., Zhang Y., Shi L., Fei X. (2022). Hypoxia-responsive aggregation of iron oxide nanoparticles for T_1_-to-T_2_ switchable magnetic resonance imaging of tumors. ACS Appl. Nano Mater..

[30] He Y., Mao Z., Lu Z., Yan J., Zhang Y., Bianco A., Cao Y., Pei R. (2022). Extremely small iron oxide nanoparticles with pH-dependent solubility transition as T_1_/T_2_ switchable contrast agents for MRI. ACS Appl. Nano Mater..

[31] Wang Z., Zhou P., Li Y., Zhang D., Chu F., Yuan F., Pan B., Gao F. (2024). A bimetallic polymerization network for effective increase in labile iron pool and robust activation of cGAS/STING induces ferroptosis-based tumor immunotherapy. Small.

[32] Li R., Zhao W., Han Z., Feng N., Wu T., Xiong H., Jiang W. (2024). Self-cascade nanozyme reactor as a cuproptosis inducer synergistic inhibition of cellular respiration boosting radioimmunotherapy. Small.

[33] Wang X., Guo Z., Zhang C., Zhu S., Li L., Gu Z., Zhao Y. (2020). Ultrasmall BiOI quantum dots with efficient renal clearance for enhanced radiotherapy of cancer. Adv. Sci..

[34] Dai Y., Zhu L., Li X., Zhang F., Chen K., Jiao G., Liu Y., Yang Z., Guo Z., Zhang B., Shen Q., Zhao Q. (2024). A biomimetic cuproptosis amplifier for targeted NIR-II fluorescence/photoacoustic imaging-guided synergistic NIR-II photothermal immunotherapy. Biomaterials.

[35] Xin H., Yuan P., Wang Y., Xiao J., Tian G., Fan Y., Zhang G., Liu L. (2024). Highly selective and effective ferroptosis liposomal nanodrugs for synergistic antitumor therapy. Chem. Eng. J..

[36] Tan M., Gao Z., Wang X., Lin C., Wan Y., Xie W., Chen W., Zhang Y., Quan Z., Hou Z. (2024). MnO_2_ nanozyme with lanthanide-based radiosensitization for advanced radiotherapy by tumor microenvironment triggering STING pathway activation. Chem. Eng. J..

[37] Liu Y., Pi F., He L., Yang F., Chen T. (2024). Oxygen vacancy-rich manganese nanoflowers as ferroptosis inducers for tumor radiotherapy. Small.

[38] Wang L., Zhang T., Huo M., Guo J., Chen Y., Xu H. (2019). Construction of nucleus-targeting iridium nanocrystals for photonic hyperthermia-synergized cancer radiotherapy. Small.

[39] Cao Y., Tang L., Fu C., Yin Y., Liu H., Feng J., Gao J., Shu W., Li Z., Zhu Y., Wang W. (2024). Black phosphorus quantum dot loaded bioinspired nanoplatform synergized with a PD-L1 for multimode cancer immunotherapy. Nano Lett..

[40] He Z., Yan H., Zeng W., Yang K., Rong P. (2021). Tumor microenvironment-responsive multifunctional nanoplatform based on MnFe_2_O_4_-PEG for enhanced magnetic resonance imaging-guided hypoxic cancer radiotherapy. J. Mater. Chem. B.

[41] Jin Y., Li D., Zheng X., Gao M., Wang W., Zhang X., Kang W., Zhang C., Wu S., Dai R., Zheng Z., Zhang R. (2024). A novel activatable nanoradiosensitizer for second near-infrared fluorescence imaging-guided safe-dose synergetic chemo-radiotherapy of rheumatoid arthritis. Adv. Sci..

[42] Lv S., Qiu Z., Yu D., Wu X., Yan X., Ren Y., Huang Y., Jiang G., Gao F. (2023). Custom-made piezoelectric solid solution material for cancer therapy. Small.

[43] Zhang M., Chen Y., Wang Q., Li C., Yuan C., Lu J., Luo Y., Liu X. (2024). Nanocatalytic theranostics with intracellular mutual promotion for ferroptosis and chemo-photothermal therapy. J. Colloid Interface Sci..

