# Supplementary material for: Janus Ag/Fe-HfO2 nanoparticles for enhanced radio-photothermal tumor therapy via magnetic resonance imaging and multienzyme activity
Source: Mater Today Bio. 2025 Jul 11;33:102083. doi: 10.1016/j.mtbio.2025.102083 (PMC12281546; doi:10.1016/j.mtbio.2025.102083)
Supplement: Multimedia component 1 [file mmc1.docx]

**Supporting Information**

**Janus Ag/Fe-HfO_2_ nanoparticles for enhanced radio-photothermal tumor therapy via magnetic resonance imaging and** **multienzyme activity**

**Xinying Liu ^a^, Baohui Liu ^a^, Conglong Chen ^a^, Chaowei Hong ^a^, Jia Xu ^a^, Yixuan Ruan ^b^, Ling Huang ^a^, Shanni Hong ^c,^ *, Wei Chen ^a,^ *, Ye Kuang ^a,^ ***

^a^ Fujian Key Laboratory of Drug Target Discovery and Structural and Functional Research, School of Pharmacy, Fujian Medical University, Fuzhou 350122, China

^b^ School of Basic Medical Sciences, Fujian Medical University, Fuzhou 350122, China.

^c^ School of Medical Imaging, Fujian Medical University, Fuzhou 350122, China.

*Corresponding authors at: School of Pharmacy, Fujian Medical University, Fuzhou 350122, China (W. Chen, Y. Kuang); School of Medical Imaging, Fujian Medical University, Fuzhou, 350122, China (S. Hong)

E-mail addresses: snhong2020@fjmu.edu.cn (S. Hong), WeiChen@fjmu.edu.cn (W. Chen), yekuang2019@fjmu.edu.cn (Y. Kuang)


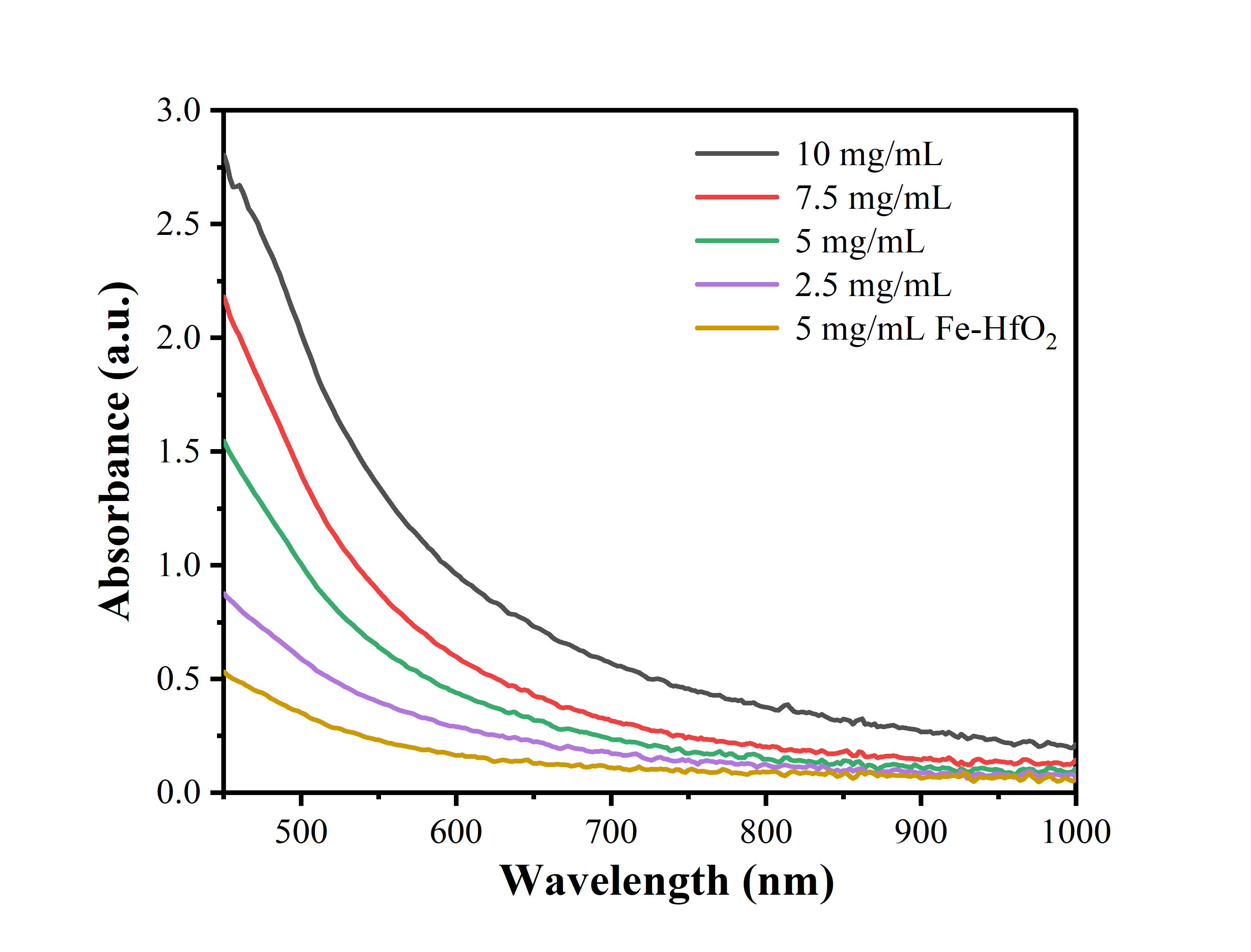


**Fig. S1.** UV-vis spectral scanning of Ag/Fe-HfO_2_ and Fe-HfO_2_ NPs at different concentrations.


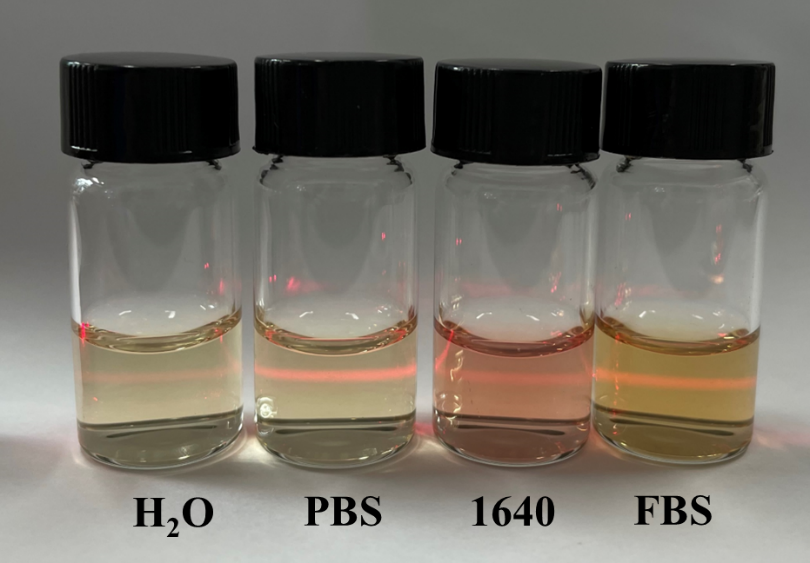


**Fig. S2.** Tyndall effect of Ag/Fe-HfO_2_-PEG-RGD solution (200 μg/mL) in H_2_O, PBS, 1640, and FBS buffer.


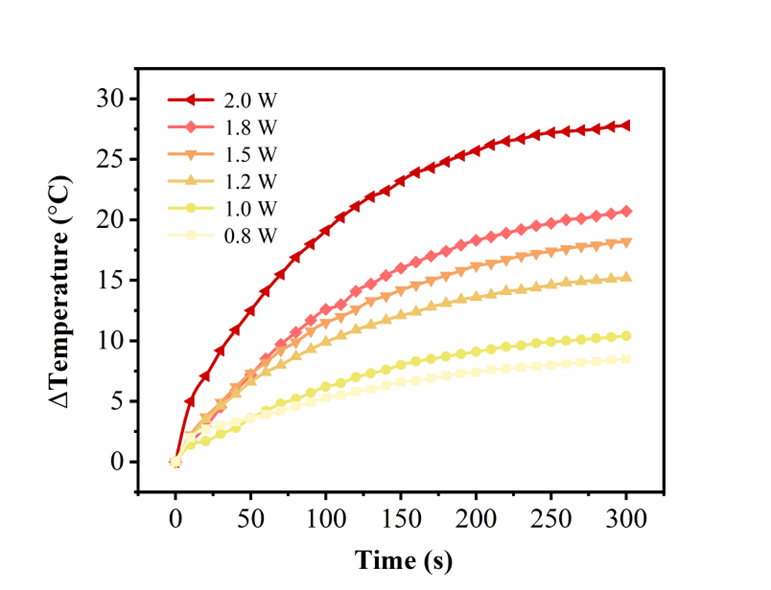


**Fig. S3.** Heating performance test of Ag/Fe-HfO_2_ NPs at different power levels.





**Fig. S4.** Photothermal profile of Ag/Fe-HfO_2_ NP_S_ in deionized water irradiated by 808 nm laser for 6 min, followed by natural cooling to room temperature (n=3).





**Fig. S5.** DMPO/·OH ESR spectra of Ag/Fe-HfO_2_ NPs and the control group.


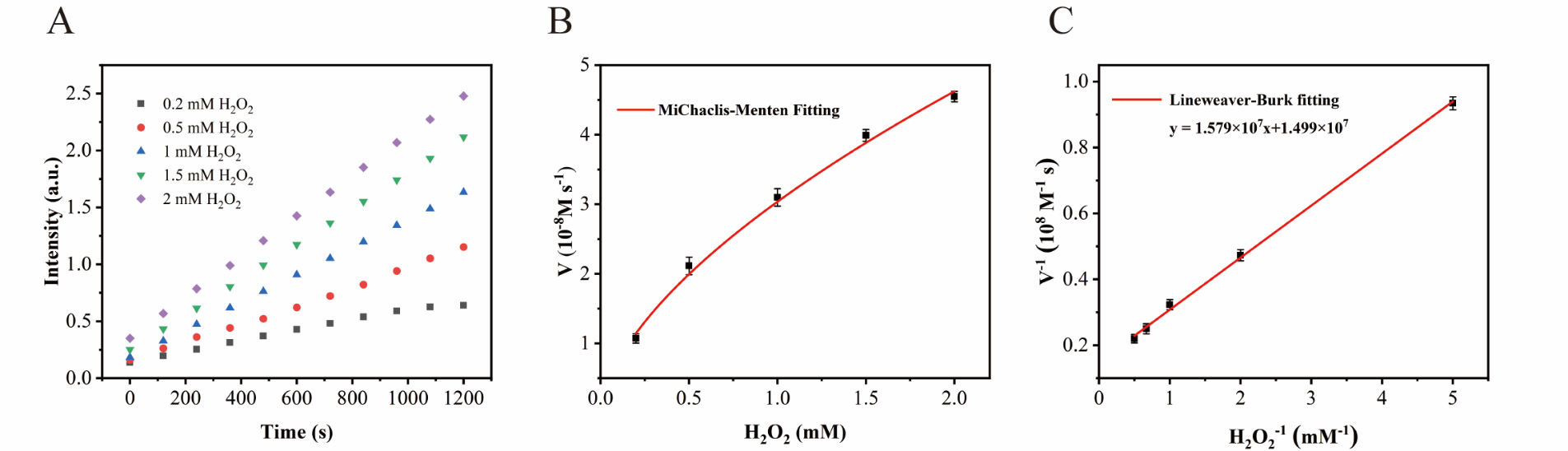


**Fig. S6.** (A) The 652 nm absorbance of TMB under conditions of Ag/Fe-HfO_2_ NPs and diverse concentrations of H_2_O_2_. (B) Michaelis-Menten fitting curve of Ag/Fe-HfO_2_ NPs at different H_2_O_2_ concentrations, n=3. (C) Lineweaver-Burk fitting curve of Ag/Fe-HfO_2_ NPs at different H_2_O_2_ concentrations, n=3.





**Fig. S7.** Relative cell viability of HC11 cells after cultivated with Janus Ag/Fe-HfO_2_ NPs and Ag/Fe-HfO_2_-PEG-RGD. Data were given as mean ± S.D. (n = 3).


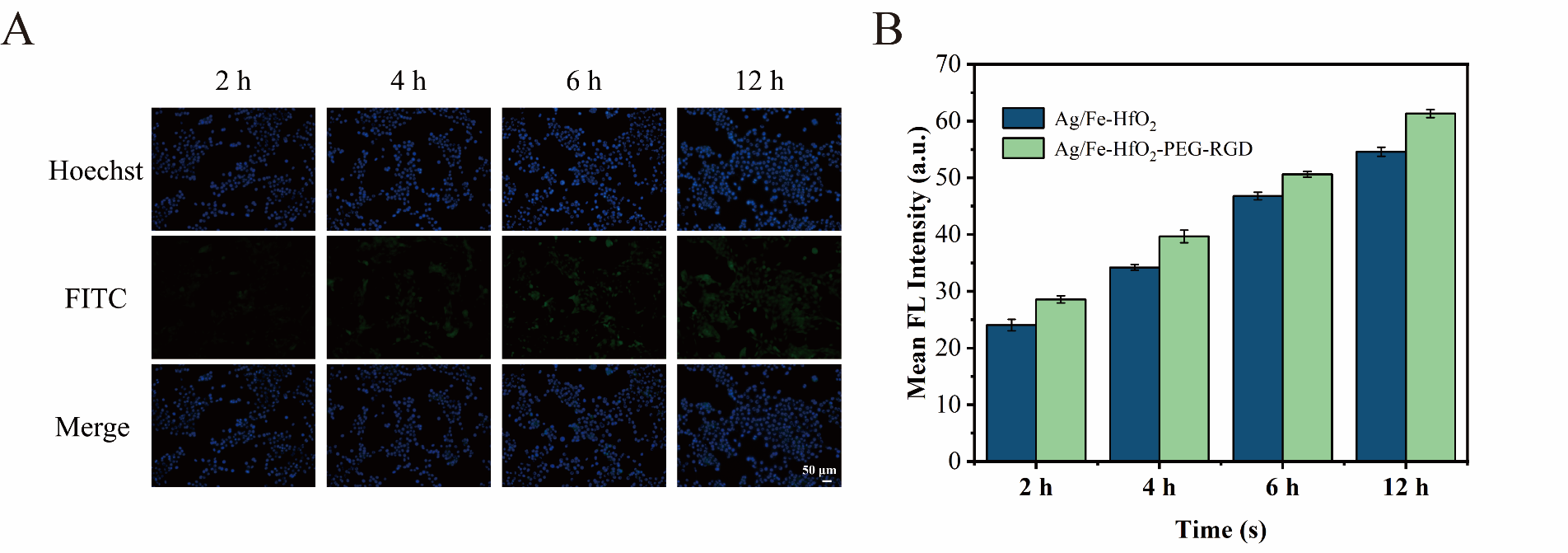


**Fig. S8.** (A) Cellular uptake of 4T1 cells incubated with FITC-labeled Ag/Fe-HfO_2_ NPs for 2, 4, 6, and 12 h. Scale bar = 50 μm. (B) The quantitative analysis of Ag/Fe-HfO_2_ NPs and Ag/Fe-HfO_2_-PEG-RGD uptake by 4T1 cells, n=3.


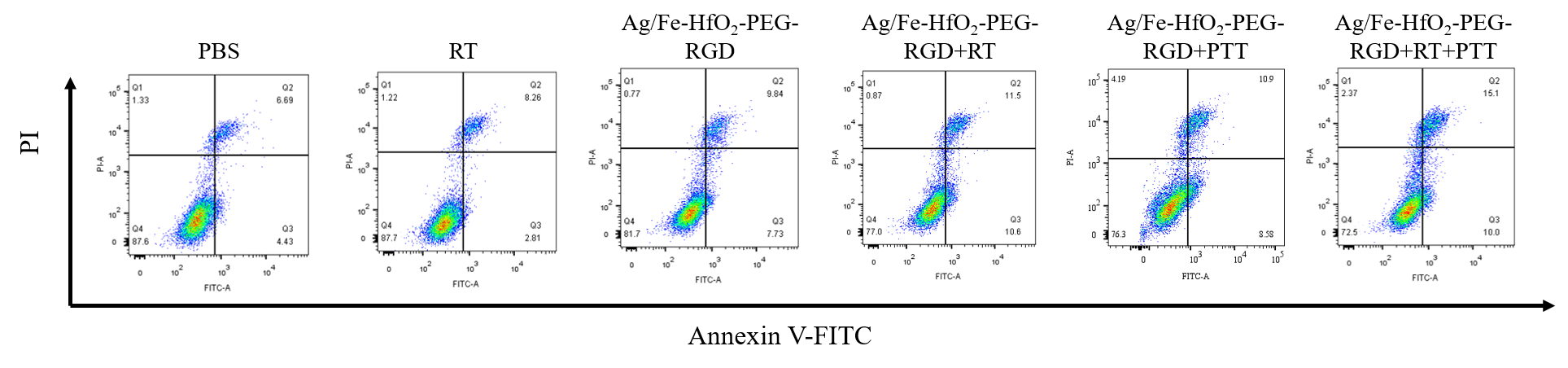


**Fig. S9.** Apoptosis flow cytometry analysis (Annexin V/PI) of 4T1 cells in different treatment groups.


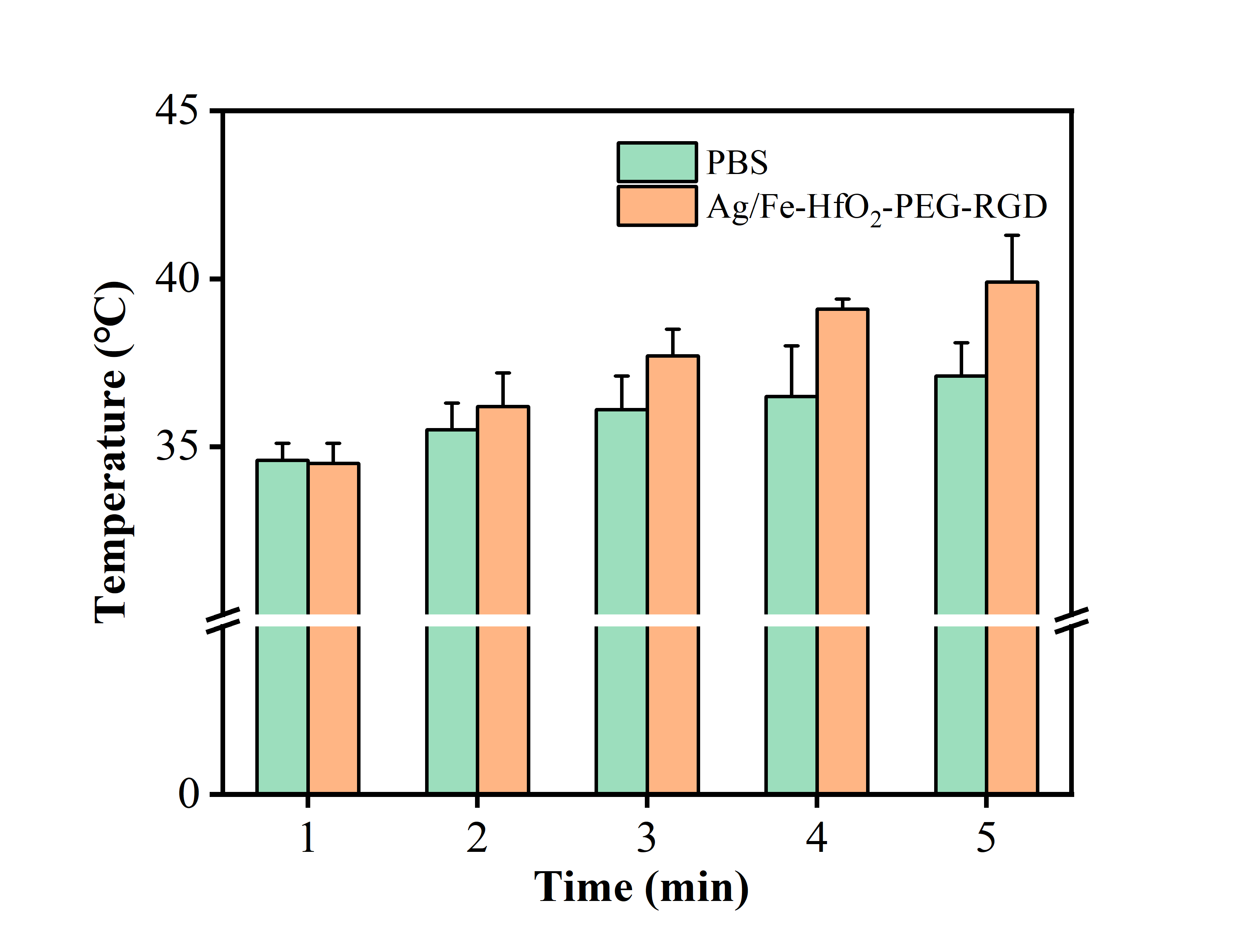


**Fig. S10.** The changes of body temperature of mice after injection of PBS or Ag/Fe-HfO_2_-PEG-RGD in tail vein with 808 nm laser at different time.
